# Genetic architecture of head rice and rice chalky grain percentages using genome-wide association studies

**DOI:** 10.3389/fpls.2023.1274823

**Published:** 2023-11-17

**Authors:** Darlene L. Sanchez, Stanley Omar PB. Samonte, Lloyd T. Wilson

**Affiliations:** Texas A&M AgriLife Research Center, Beaumont, TX, United States

**Keywords:** rice, milled rice, head rice, chalky rice, grain quality, genome-wide association study

## Abstract

High head rice and low chalky grain percentages are key grain quality traits selected in developing rice cultivars. The objectives of this research were to characterize the phenotypic variation of head rice and chalky grain percentages in a diverse collection of rice accessions, identify single nucleotide polymorphism (SNP) markers associated with each of these traits using genome-wide association studies (GWAS), and identify putative candidate genes linked to the SNPs identified by GWAS. Diverse rice varieties, landraces, and breeding lines were grown at the Texas A&M AgriLife Research Center in Beaumont. Head rice percentages (HRP) and chalky grain percentages (CGP) of 195 and 199 non-waxy accessions were estimated in 2018 and 2019, respectively. Phenotypic data were analyzed along with 854,832 SNPs using three statistical models: mixed linear model (MLM), multi-locus mixed model (MLMM), and fixed and random model circulating probability unification (FarmCPU). Significant variations in HRP and CGP were observed between rice accessions. Two significant marker-trait associations (MTAs) were detected on chromosomes 1 and 2, respectively, based on best linear unbiased prediction (BLUP) values in 2018, while in 2019, one SNP was significantly associated with HRP in each of chromosomes 6, 8, 9, and 11, and two in chromosome 7. CGP was significantly associated with five SNPs located in chromosomes 2, 4, 6, and 8 in the 2018 study and ten SNPs in chromosomes 1, 2, 3, 4, 7, 8, 11, and 12 in the 2019 study. The SNPs are located within or linked to putative candidate genes involved in HRP and CGP. This study reports five and ten novel MTAs for HRP and CGP, respectively, while three and five MTAs co-located with previously reported quantitative trait loci for HRP and CGP, respectively. The validation of candidate genes for their roles in determining HRP and CGP is necessary to design functional molecular markers that can be used to effectively develop rice cultivars with desirable grain quality.

## Introduction

High grain quality is an important selection criterion in developing rice cultivars. Head rice percentage (HRP), defined as the proportion of whole milled grains to paddy rice, and chalky grain percentage (CGP), defined as the proportion of grains having at least 50% whitish area, greatly influence the market value of rice (Fitzgerald et al., 2009; Bao, 2019; Liu et al., 2020).

Head rice and chalkiness are complex traits that are influenced by multiple genes or cultivar differences and by environmental conditions that occur during the crop’s growth, harvesting, milling, drying, and storage, and by post-harvest processes (Liu et al., 2010; Chen et al., 2013; Sreenivasulu et al., 2015; Zhou et al., 2015; Bao, 2019). Head rice can be influenced by genetic variation within the *Waxy* gene (Deng et al., 2022), as well as differences in rice grain protein composition (Balindong et al., 2018). An increase of 1°C in the mean temperature during the growing season can reduce head rice by 9.0 to 13.8% (Lyman et al., 2013). The degree of reduction in head rice and increase in chalky grains due to increased nighttime temperatures varies between rice genotypes (Counce et al., 2005; Cooper et al., 2008; Lanning et al., 2011; Lanning and Siebenmorgen, 2013). Head rice yield is positively associated, and chalkiness negatively associated with relative humidity, while the reverse relationships apply to both traits and vapor pressure deficit (Zhao and Fitzgerald, 2013; Xia et al., 2021). Rainfall or sudden increase in relative humidity following a period of low relative humidity can cause fissuring in rice, which reduces head rice (Jodari and Linscombe, 1996). In contrast, nitrogen fertilization has been shown to increase head rice and chalky grain percentages (Rogers et al., 2016; Wada et al., 2019).

A number of quantitative trait loci (QTLs) for HRP (Aluko et al., 2004; Dong et al., 2004; Li et al., 2004; Zheng et al., 2007; Kepiro et al., 2008; Nelson et al., 2011; Nelson et al., 2012; Zhang et al., 2020) and CGP (Zhou et al., 2009; Mei et al., 2013; Bian et al., 2014; Peng et al., 2014; Gao et al., 2016; Zhao et al., 2016) have been identified using biparental mapping populations. Genome-wide association studies (GWAS) use diverse germplasm and high-density markers in QTL mapping. In contrast to biparental crosses, which utilize recent recombination events that took place during the development of the mapping population, a diverse collection of germplasm encapsulates all historical recombination events that occur during the evolution of each accession (Lipka et al., 2015). Genomic regions and candidate genes associated with HRP and CGP have been mapped using association mapping (Qiu et al., 2015; Quero et al., 2018; Misra et al., 2019; Liu et al., 2020; Misra et al., 2021; Xu et al., 2022; Huo et al., 2023).

A diverse panel of rice accessions, composed of 71.4% *japonica*, 8.2% *indica*, 0.9% *aus*, and 19.5% admixed (Alpuerto et al., 2022; Sanchez et al., 2022) were used for association mapping of head rice and chalky grain percentages. The objectives were to describe the phenotypic variation of head rice and chalky grain percentages in diverse rice accessions, identify single nucleotide polymorphism (SNP) markers associated with these traits, and identify putative candidate genes linked to significant marker-trait associations.

## Materials and methods

### Plant materials and field experiment set-up

**Figure 1:**
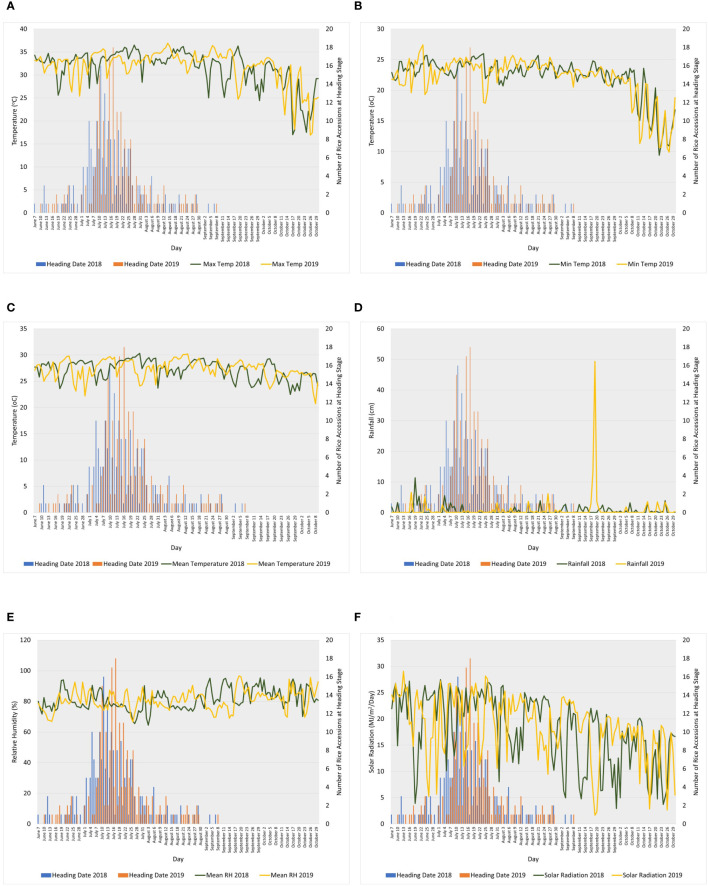
Line graphs showing daily **(A)** maximum temperature, **(B)** minimum temperature, **(C)** mean temperature, **(D)** rainfall, **(E)** relative humidity, and **(F)** solar radiation during heading to maturity stages (shown in bar graphs) of the association panel in 2018 and 2019 at Texas A&M AgriLife Research Center in Beaumont. Weather data source: iAIMS Climatic Data of Texas A&M AgriLife Research Center in Beaumont. https://beaumont/tamu.edu/ClimaticData/ (Wilson et al., 2007; Yang et al., 2010; Wilson et al., 2015).

Rice accessions consisting of a diverse set of *indica* and *japonica* cultivars, landraces, inbred lines, and hybrids (Alpuerto et al., 2022; Sanchez et al., 2022) were planted at the Texas A&M AgriLife Research Center in Beaumont in 2018 and 2019. Daily temperature (maximum, minimum, and mean), rainfall, relative humidity, and solar radiation at the research center are shown in **Figure 1** (Wilson et al., 2007; Yang et al., 2010; Wilson et al., 2015). A total of 217 and 207 rice accessions were analyzed for HRP in 2018 and 2019, respectively. Waxy or glutinous rice accessions, which have typically opaque endosperm, were excluded in the CGP analyses; hence, 195 and 199 entries were analyzed for CGP in 2018 and 2019, respectively.

The field experiments in both years were laid out in four blocks using an augmented design, with five checks (Antonio, Cheniere, Cocodrie, Colorado, and Presidio) replicated in each block. Each rice accession was drill-seeded into three-row plots that were 2.44 m long, with rows spaced 0.28 m apart. Planting was done on April 19, 2018, and April 16, 2019. In 2018, nitrogen was applied as urea in two splits, 108 kg ha^−1^ N at planting and 129 kg ha^−1^ N at 6 weeks after planting. In 2019, urea was applied in three splits: 59 kg N ha^−1^ at planting, 129 kg N ha^−1^ at two weeks after planting, and 47 kg N ha^−1^ at 11 weeks after planting. In both years, the fields were flushed irrigated after drill-seeding and as needed within one month after sowing. Permanent flood was maintained at a 10-cm water depth starting one month after planting.

### Phenotyping for head rice and chalky grain percentages

Paddy rice samples of each accession were harvested 30–35 days after heading. Samples were threshed and oven-dried at 38°C until the grain moisture was about 14%. A 200-g sample of rice from each accession was used for head rice determination. All samples were dehulled using a Yamamoto FC2K-Y testing husker (Yamamoto Co. Ltd., Yamagata-ken, Japan) and milled using a Zaccaria PAZ 1 DTA mill (Zaccaria USA, Anna, TX, USA). The milled rice samples were weighed to determine the total milled rice and a Zaccaria CRZ 5 (ZaccariaUSA, Anna, TX, USA) was used to separate the milled rice into whole and broken grain. The whole grain was weighed to determine the head rice percentage. The chalky grain percentage was measured using 50 g of whole milled rice grain in the S21 Rice Statistical Analyzer (TKD Tecnologia, Brazil). A grain is considered chalky if its whitish area is at least 50%. The formula used in computing for CGP is as follows:


$$CGP\;\left(\%\right)\;=\frac{The\;number\;of\;grains\;with\;at\;least\;50\%\;whitish\;area}{Total\;number\;of\;grains}\;\times 100\%$$

### Marker data

The SNP markers used in this study were generated using genotyping-by-sequencing with 1X coverage, performed at the Texas A&M AgriLife Genomics and Bioinformatics Service (TxGen). The reference genome used was the International Rice Genome Sequencing Project (IRGSP) Build 5 Pseudomolecules of the Rice Genome, *Oryza sativa* ssp. *japonica* cultivar Nipponbare (Kawahara et al., 2013). The raw marker data was filtered, in which SNPs with more than 50% missing data and minimum allele frequency (MAF) of less than 5% were eliminated, bringing the initial number of SNPs to 1,075,302. Imputation was conducted using BEAGLE V4.0 (Browning and Browning, 2007). The imputed genotype data was filtered a second time using TASSEL 5.2.61 (Bradbury et al., 2007), where SNPs with less than 5% MAF and more than 5% missing data were removed, resulting in 854,832 SNPs that were used in the association analyses. The marker data is available on Dryad (https://doi.org/10.5061/dryad.4qrfj6qbs, Sanchez et al., 2022).

### Data analyses

Analysis of variance (ANOVA) was conducted using SAS Version 9.4 (SAS 2016). The PROC MIXED in SAS software was used to estimate fixed and random effects, and these were used to estimate best linear unbiased prediction (BLUP) in R version 3.6.1 (R Core Team, 2021). ANOVA and broad-sense heritability (H^2^) for HRP and CGP for each year were estimated using the ‘augmentedRCBD’ package in R (Aravind et al., 2020). Pearson’s pairwise correlation coefficients were also calculated using R.

GWAS for HRP and CGP were conducted using the phenotype data for 2018 and 2019 separately, as well as the BLUPs estimates from both years. Factors that may cause false trait-SNP associations [i.e., population structure (Q) and genetic relatedness (K)] were controlled using principal component analysis (PCA) and kinship matrix, respectively.

The PCA kinship matrix (VanRaden, 2008) and linkage disequilibrium (LD) decay have been determined in previous studies that used the same population as used in this experiment (Alpuerto et al., 2022; Sanchez et al., 2022). The first four principal components (PC) explained 56.7% of the total genetic variation. The first PC explained 38.9% of the variation and distinguished the *indica* from the *japonica rice*. The second PC, explaining 12.0% of the variation, further divided the *japonicas* into *temperate japonica* and *tropical japonica* subpopulations. Rice accessions classified as admixed were found between these three major subgroups (Alpuerto et al., 2022; Sanchez et al., 2022). LD decay, the distance where the mean r^2^ decreased to half its maximum value, was calculated using TASSEL 5.2.61 (Bradbury et al., 2007) to estimate the appropriate resolution for association mapping. The average genome-wide LD decay of the population has been estimated to be at about 150,000 bp (Alpuerto et al., 2022; Sanchez et al., 2022).

Association analyses were conducted using the mixed linear model (MLM) (Yu et al., 2006), multi-locus mixed model (MLMM) (Segura et al., 2012), and fixed and random model circulating probability unification (FarmCPU) (Liu et al., 2016), implemented in GAPIT Version 3 (Wang & Zhang, 2021). All three models include Q and K to account for false positives. MLMM uses a stepwise linear mixed-model regression that includes significantly associated markers as cofactors, while FarmCPU includes additional algorithms to solve the confounding problems between testing markers and covariates. The multiple testing threshold to declare significant SNP-trait associations was set to *p* = 2.91 × 10^−7^ (Sanchez et al., 2022) based on results from the statistical program ‘SimpleM’ (Gao et al., 2010; Johnson et al., 2010). The R package ‘CMplot’ (Yin et al., 2021) was used to construct the circular Manhattan plots. Allelic effects of significant marker-trait associations were analyzed using JMP ver. 14 software (SAS Institute). Identification of genes that contain the significant SNPs was achieved using the Nipponbare IRGSP Build 5 genome browser in the Rice Annotation Project Database (RAP-DB) (https://rapdb.dna.affrc.go.jp/viewer/gbrowse/build5/) (Sakai et al., 2013). Haplotype analyses for selected gene models were conducted using the R package ‘geneHapR’ (Zhang et al., 2023).

## Results

### Variation in head rice and chalky grain percentages

The rice accessions exhibited wide variation in HRP and CGP (**Table 1**, **Table S1**). HRP ranged from 30.7 (Khao Phoi) to 68.6% (Taichu Mochi 59) in 2018 and from 6.0 (Chia Nung Yu 242) to 73.4% (RU-1603126) in 2019. CGP ranged from 0.0 (Palmyra) to 46.8% (WIR 3039) in 2018 and from 0.18 (IR 1321-12) to 42.4% (Baber) in 2019. Presidio, one of the check cultivars, had an average of 62.3% HRP and 0.7% CGP in 2018, and an average of 58.8% HRP and 0.7% CGP in 2019. Two rice accessions consistently had the lowest chalky grain percentage in both years: IR 1321-12 (0.1% in both 2018 and 2019) and Palmyra (0.0% in 2018 and 0.1% in 2019). In terms of head rice percentage, Kamenoo (66.6% in 2018 and 69.2% in 2019) and N22 (67.6% in 2018 and 69.3% in 2019) were consistently in the top ten, while Khao Phoi (30.7% in 2018 and 36.7% in 2019), R 67 (35.3% in 2018 and 16.2% in 2019), and 172R (39.2% in 2018 and 43.8% in 2019) were consistently in the bottom ten. Broad-sense heritability (H^2^) estimates were high for both traits. The H^2^ estimate for HRP was 0.96 in both years, while CGP had H^2^ estimates of 0.98 and 0.99 in 2018 and 2019, respectively.

**Table 1:** Summary statistics, broad-sense heritability (H^2^) estimates, and check values for head rice and chalky grain percentages of rice accessions grown in Texas A&M AgriLife Research at Beaumont in 2018 and 2019.

Statistics	Head Rice Percentage (%)		Chalky Grain Percentage (%)	
	2018	2019	2018	2019
Mean	56.5	59.2	12.2	6.7
Standard Deviation	8.1	9.1	11.4	8.4
Minimum	30.7	6.0	0.0	0.1
Maximum	68.6	73.4	46.8	42.4
Broad-sense Heritability (H^2^)	0.96	0.96	0.98	0.99
Checks				
Antonio	63.7	57.6	4.0	3.4
Cheniere	64.0	57.6	1.6	1.0
Cocodrie	64.6	62.0	3.5	2.3
Colorado	52.9	57.1	4.5	0.9
Presidio	62.3	58.8	0.7	0.7

**Figure 2:**
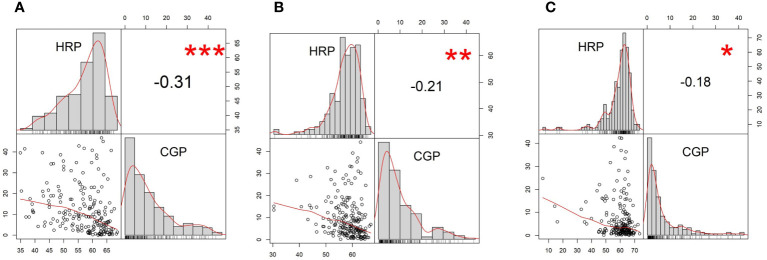
Frequency distribution and pairwise correlations of head rice percentage (HRP) and chalky grain percentage (CGP) from **(A)** best linear biased prediction (BLUP), **(B)** 2018, and **(C)** 2019. The upper left and lower right quadrants show the histograms of the two traits. Shown in the lower left quadrants are pairwise scatterplots. Shown in the upper right quadrants are the pairwise Pearson correlation coefficients among HRP and CGP. ***Significant at *p* = 0.001; **Significant at *p* = 0.01; *Significant at *p* = 0.05.

Best linear unbiased prediction (BLUP) values for HRP followed a negatively skewed distribution, while those of CGP followed a positively skewed distribution (**Figure 2A**). HRP and CGP had a weak, negative correlation (Pearson correlation coefficient (r) = −0.21) based on the BLUPs estimated from the two-years’ data (**Figure 2A**). Frequency distribution trends and correlation coefficients for HRP and CGP were similar in 2018 and 2019 (**Figures 2B, C**, respectively). HRP and CGP also had low negative correlations in 2018 (r = −0.31) (**Figure 2B**) and 2019 (r = −0.18) (**Figure 2C**).

Analysis of variance showed highly significant variation due to year-by-genotype interaction effects (*p *< 0.0001) in both HRP and CGP (**Table 2**). Significant differences in HRP due to genotypes and checks were also observed, while CGP was significantly different between years, genotypes, and checks. There was no evidence of significant variation in HRP and CGP between blocks within the years 2018 and 2019.

**Table 2:** Analyses of variance for head rice and chalky grain percentages of rice accessions grown in Texas A&M AgriLife Research at Beaumont in 2018 and 2019.

Trait: Head Rice Percentage						
Source of Variation	DF	Type III SS	Mean Square	F Value	Pr > F	
Year	1	6.99	6.99	0.45	0.5096	
Block(Year)	6	25.92	4.32	0.28	0.9429	
Check	4	263.65	65.91	4.22	0.0092	**
Genotype	214	19536.74	91.29	5.84	<0.0001	***
Year x Genotype	199	11233.24	56.44	3.61	0.0001	***
Trait: Chalky Grain Percentage						
Source of Variation	DF	Type III SS	Mean Square	F Value	Pr > F	
Year	1	53.34	53.34	22.57	<0.0001	***
Block(Year)	6	6.95	1.16	0.49	0.8097	
Check	4	90.35	22.59	9.56	<0.0001	***
Genotype	202	30137.33	149.19	63.13	<0.0001	***
Year x Genotype	181	7397.36	40.87	17.29	<0.0001	***

Levels of significance: **p < 0.01; ***p < 0.001.

### Genome-wide association studies and identification of candidate genes for HRP and CGP

GWAS was conducted using 854,832 SNPs and BLUP estimates for HRP and CGP. Because there was a significant effect of year on both traits (**Table 2**), GWAS were also conducted separately for the 2018 and 2019 data.

**Figure 3:**
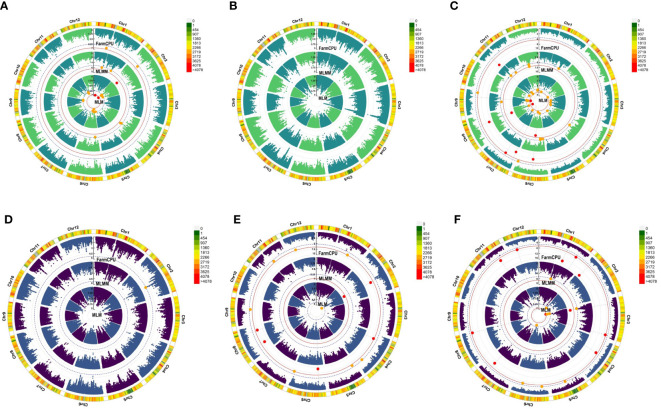
Manhattan plots showing significant (red dots) and suggestive (orange dots) SNP marker-trait associations (MTAs) with head rice percentage (HRP) in **(A)** BLUP estimates, **(B)** 2018, and **(C)** 2019 and with chalky grain percentage (CGP) in **(D)** BLUP estimates, **(E)** 2018, and **(F)** 2019. The models used to detect these MTAs are in the following order from innermost to outermost circle: mixed linear model (MLM), multi-locus mixed model (MLMM), and fixed and random model circulating probability unification (FarmCPU). The red solid line marks the significance threshold (*p* = 2.91 x 10^−7^), and the blue broken line marks the suggestive threshold (*p* = 5.83 x 10^−6^).

Twenty-three significant marker-trait associations (MTAs) were identified (**Table 3**, **Figure 3**). In the BLUPs estimated from the 2018 and 2019 data, HRP was significantly associated with two SNPs (**Figure 3A**). The top SNP marker was S02_10354220, located in chromosome 2, and was detected in the MLM (*p* = 1.54 × 10^−7^) and MLMM (*p* = 2.02 × 10^−8^). The other marker significantly associated with HRP was S01_14089465 in chromosome 1 and was detected by the MLM (*p* = 2.14 × 10^−7^). The top SNP in the 2018 study was S03_32703779 (*p* = 1.55 × 10^−5^), located on chromosome 3 (**Figure 3B**). Six SNPs in chromosomes 6, 7, 8, 9, and 11 were significantly associated with HRP in 2019 (**Figure 3C**). All three models detected S08_18460399, while S06_14934656 was detected by FarmCPU and MLMM.

**Table 3:** Single nucleotide polymorphism markers significantly associated with head rice percentage (HRP) and chalky grain percentage (CGP) in rice accessions grown in Texas A&M AgriLife Research at Beaumont in 2018 and 2019.

Trait	SNP	Alleles	Chromosome	Position (bp)	Experiment	Model	*p* value	−Log10 (p)	Allelic Effect^a^	Previously reported QTL for Head Rice and Chalkiness
**HRP (%)**	S01_14089465	A/G	1	14,089,465	BLUP	MLM	2.14E-07	6.7	6.1	*hr1* (Aluko et al., 2004)
	S02_10354220	C/T	2	10,354,220	BLUP	MLM	1.54E-07	6.8	−6.6	
						MLMM	2.02E-08	7.7		
	S03_32703779^b^	A/G	3	32,703,779	2018	FarmCPU	1.55E-05	4.8	6.1	*qHR3* (Zhang et al., 2020)
	S06_14934656	A/G	6	14,934,656	2019	FarmCPU	2.80E-09	8.6	−6.7	
						MLMM	3.99E-08	7.4		
	S07_1300806	G/T	7	1,300,806	2019	FarmCPU	8.44E-11	10.1	5.8	*qHR-7* (Li et al., 2004)
	S07_11468500	A/G	7	11,468,500	2019	FarmCPU	1.59E-07	6.8	−4.9	
	S08_18460399	C/T	8	18,460,399	2019	FarmCPU	9.26E-12	11.0	−7.7	
						MLM	3.73E-08	7.4	−9.5	
						MLMM	2.48E-10	9.6		
	S09_17570598	A/T	9	17,570,598	2019	MLM	1.27E-07	6.9	12.4	
	S11_8199218	A/G	11	8,199,218	2019	FarmCPU	1.19E-11	10.9	−5.8	*qHRY11* (Zhang et al., 2020)
**CGP (%)**	S01_25791807	A/C	1	25,791,807	2019	FarmCPU	1.34E-14	13.9	−6.1	*QPGWC.NH-1.1* (Bian et al., 2014)
	S01_36699691	C/T	1	36,699,691	2019	FarmCPU	1.31E-07	6.9	−3.2	
	S02_2877652	A/T	2	2,877,652	2019	FarmCPU	2.65E-16	15.6	−5.5	*qPGWC2a* (Zhao et al., 2016)
	S02_22742146^c^	A/G	2	22,742,146	BLUP	FarmCPU	5.05E-06	5.3	−5.3	
	S02_23451362	C/T	2	23,451,362	2018	FarmCPU	1.81E-08	7.7	7.7	*qPGWC2c; qDEC2b* (Zhao et al., 2016)
						MLMM	1.23E-07	6.9		
	S03_4501040	A/G	3	4,501,040	2019	MLM	2.16E-07	6.7	−11.5	
						MLMM	1.67E-08	7.8		
	S03_35171822	C/G	3	35,171,822	2019	FarmCPU	1.55E-07	6.8	−2.4	*qPGWC3b* (Zhao et al., 2016)
	S04_5091349	G/T	4	5,091,349	2018	FarmCPU	1.64E-07	6.8	3.7	
	S04_13964201	A/C	4	13,964,201	2019	FarmCPU	2.71E-08	7.6	−3.3	
	S06_6057367	A/G	6	6,057,367	2018	FarmCPU	4.88E-10	9.3	4.4	
	S07_22868199	A/G	7	22,868,199	2019	FarmCPU	1.67E-10	9.8	−4.7	
	S08_8532874	A/G	8	8,532,874	2018	FarmCPU	4.60E-09	8.3	3.3	
	S08_23740235	C/T	8	23,740,235	2018	FarmCPU	3.15E-08	7.5	8.1	
	S08_27534174	A/G	8	27,534,174	2019	FarmCPU	6.25E-08	7.2	3.6	*qDEC8b* (Zhao et al., 2016)
	S11_23264123	A/G	11	23,264,123	2019	FarmCPU	3.28E-09	8.5	−4.0	
	S12_7883496	A/G	12	7,883,496	2019	FarmCPU	1.67E-07	6.8	−3.1	

a Allelic effect estimate is with respect to the nucleotide that is second in alphabetical order.

b Top SNP in the 2018 HRP dataset.

c Top SNP in the BLUP CGP dataset.

For CGP, the top SNP in the BLUP dataset was S02_22742146 (*p* = 5.05 × 10^−6^), located on chromosome 2 (**Figure 3D**). Five SNPs in chromosomes 2, 4, 6, and 8 were significantly associated with CGP in the 2018 dataset (**Figure 3E**). Both FarmCPU and MLMM detected the SNP S02_23451362. Ten SNPs in chromosomes 1, 2, 3, 4, 7, 8, 11, and 12 were significantly associated with CGP in the 2019 dataset (**Figure 3F**), with S03_4501040 detected by both MLM and MLMM.

**Figure 4:**
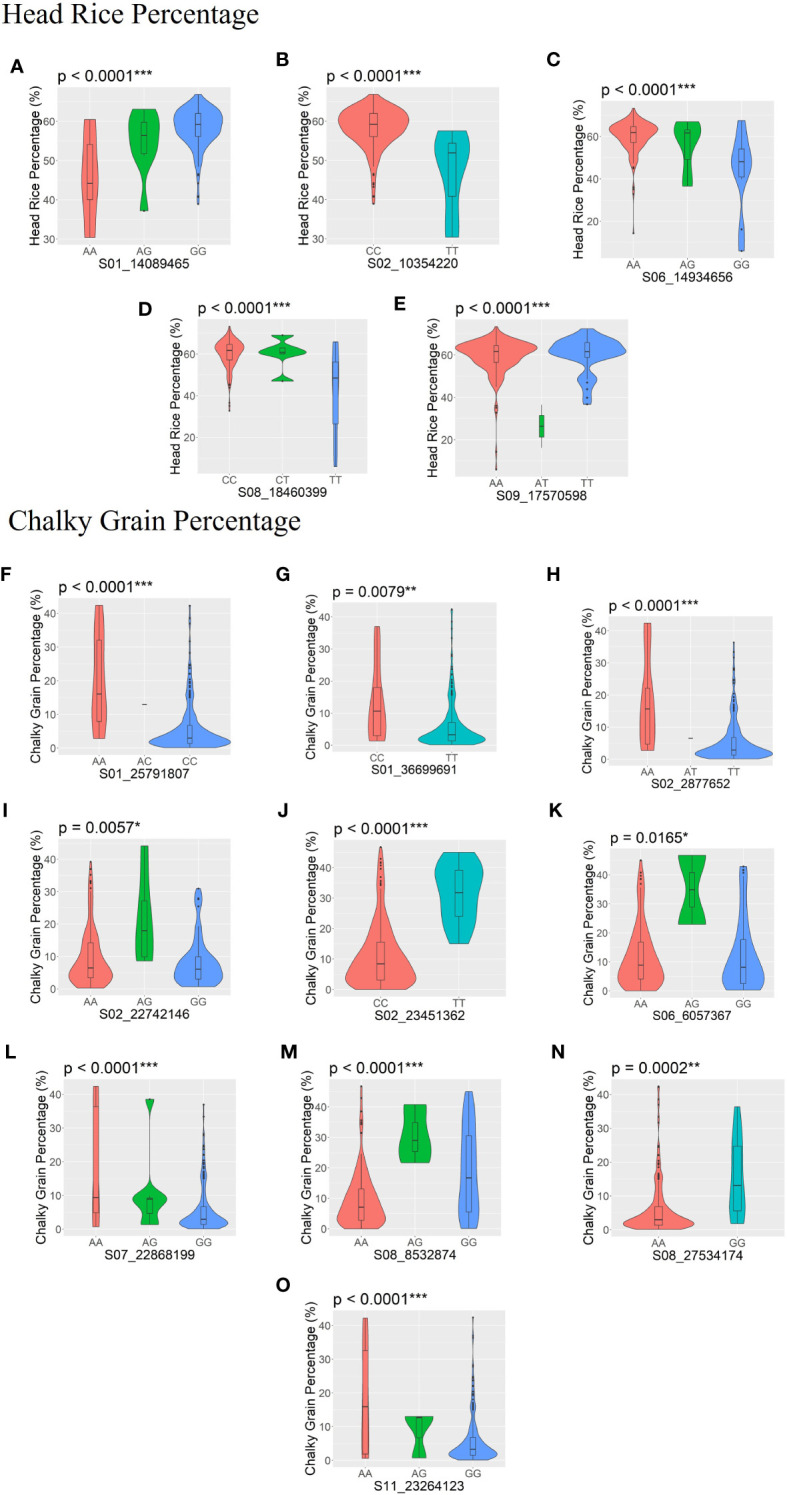
SNPs with significant allelic effects on head rice percentage (HRP; **A–E**) and chalky grain percentage (CGP; **F–O**) are shown by boxplots within violin plots. The boxplot represents the interquartile range. The central line represents the median value. The gray shape on each side of the boxplot represents all measured data points, and the thickness represents the probability density of the data. The *p*-values of the allelic effects on each SNP for HRP and CGP are shown above each small plot. *Significant at *p* ≤ 0.05; **significant at *p* ≤ 0.01; ***significant at *p* ≤ 0.001.

Significant allelic effects (*p *< 0.05) were found in five SNPs associated with HRP (S01_14089465, S02_10354220, S06_14934656, S08_18460399, and S09_17570598) (**Figures 4A-E**), while 10 SNPs were significantly associated with CGP (S01_25791807, S01_36699691, S02_2877652, S02_22742146, S02_23451362, S06_6057367, S07_22868199, S08_8532874, S08_18460399, and S09_17570598) (**Figures 4F–O**).

SNPs that have significant associations with HRP and CGP were identified using the Nipponbare IRGSP Build 5 genome browser in RAP-DB (Kawahara et al., 2013; Sakai et al., 2013) (**Table S2**). A total of 116 annotated genes were found to be in LD with the SNPs significantly associated with HRP, and 326 with the SNPs significantly associated with CGP.

Three significant MTAs from each trait were located within gene models. The three SNPs significantly associated with HRP, S07_1300806, S08_18460399, and S11_8199218, are within gene models Os07g0124700, or the *PLETHORA8* locus (Kitomi et al., 2011; Harrop et al., 2019), Os08g0388900 (similar to para-hydroxybenzoate-polyprenyltransferase), and Os11g0252400 (ankyrin repeat containing protein), respectively. The SNPs significantly associated with CGP, S01_25791807, S02_2877652, and S11_23264123, are located within the gene models Os01g0610600 (endonuclease/exonuclease/phosphatase domain-containing protein), Os02g0152500 or *VERNALIZATION INSENSITIVE 3-LIKE 2/VERNALIZATION INSENSITIVE 3-LIKE 3/Leaf Inclination 2* locus (Zhao et al., 2010; Wang et al., 2013; Yang et al., 2013), and Os11g0565660 (predicted locus).

Putative candidate genes which may have a role in head rice and chalky grain percentages were Os12g0242100 (Similar to glycine-rich cell wall structural protein 1 precursor) and Os07g0124750 (CELLULOSE SYNTHASE LIKE C10) (Kawahara et al., 2013; Sakai et al., 2013). Gene models linked to SNPs significantly associated with HRP (Os09g0451500) and CGP (Os01g0610700, Os02g0571900, Os02g0572200, Os02g0572300, Os02g0572600, Os02g0586900, Os02g0587000, Os03g0185800, Os07g0555000, Os08g0480400, and Os12g0243100) are expressed in the endosperm (**Table 4**), based on Rice Expression Profile (RiceXPro) database (Sato et al., 2013).

**Table 4:** Gene models in linkage disequilibrium to SNPs significantly associated with head rice percentage (HRP) and rice chalky grain percentage (CGP), which are expressed in the endosperm.

Trait	SNP	Locus (IRGSP Build 5)	Chromosome	Location (IRGSP Build 5)		DNA Strand Direction	Gene Product	Annotation
				Start (bp)	End (bp)			
HRP	S09_17570598	Os09g0451500	9	17636986	17641903	+	Os09t0451500-01	Similar to protein disulfide isomerase
							Os09t0451500-02	Thioredoxin domain 2 containing protein
CGP	S01_25791807	Os01g0610700	1	25794032	25796876	–	Os01t0610700-01	Zinc finger, RING/FYVE/PHD-type domain-containing protein
	S02_22742146	Os02g0571900	2	22731595	22733235	–	Os02t0571900-01	Cytochrome P450 family protein
	S02_22742146	Os02g0572200	2	22744365	22745676	+	Os02t0572200-01	Similar to RING-H2 finger protein ATL3I (YGHL1-C3HC4 RING fusion protein)
	S02_22742146	Os02g0572300	2	22749914	22750960	–	Os02t0572300-00	Similar to RING-H2 finger protein ATL3B
	S02_22742146	Os02g0572600	2	22776901	22778769	–	Os02t0572600-01	Protein kinase PKN/PRK1, effector domain-containing protein
	S02_23451362	Os02g0586900	2	23449381	23450158	+	Os02t0586900-01	Hypothetical conserved gene
							Os02t0586900-02	Similar to Glycine rich protein (Fragment)
	S02_23451362	Os02g0587000	2	23453271	23454177	+	Os02t0587000-01	Similar to Glycine rich protein (Fragment)
	S03_4501040	Os03g0185800	3	4493722	4494489	+	Os03t0185800-01	Conserved hypothetical protein
	S07_22868199	Os07g0555000	7	22767847	22770288	+	Os07t0555000-01	F-box domain, Skp2-like domain-containing protein
							Os07t0555000-02	F-box domain protein, regulation of seed development (OsFbox394)
	S08_23740235	Os08g0480400	8	23818719	23820298	–	Os08t0480400-01	Similar to cupin, RmlC-type
	S12_7883496	Os12g0242100	12	7881791	7882754	–	Os12t0242100-01	Similar to Glycine-rich cell wall structural protein 1 precursor
	S12_7883496	Os12g0243100	12	7943410	7943793	+	Os12t0243100-00	Hypothetical conserved gene

Expression data from Rice XPro Database (Sato et al., 2013). +, Leading strand; –, Lagging strand.

**Figure 5:**
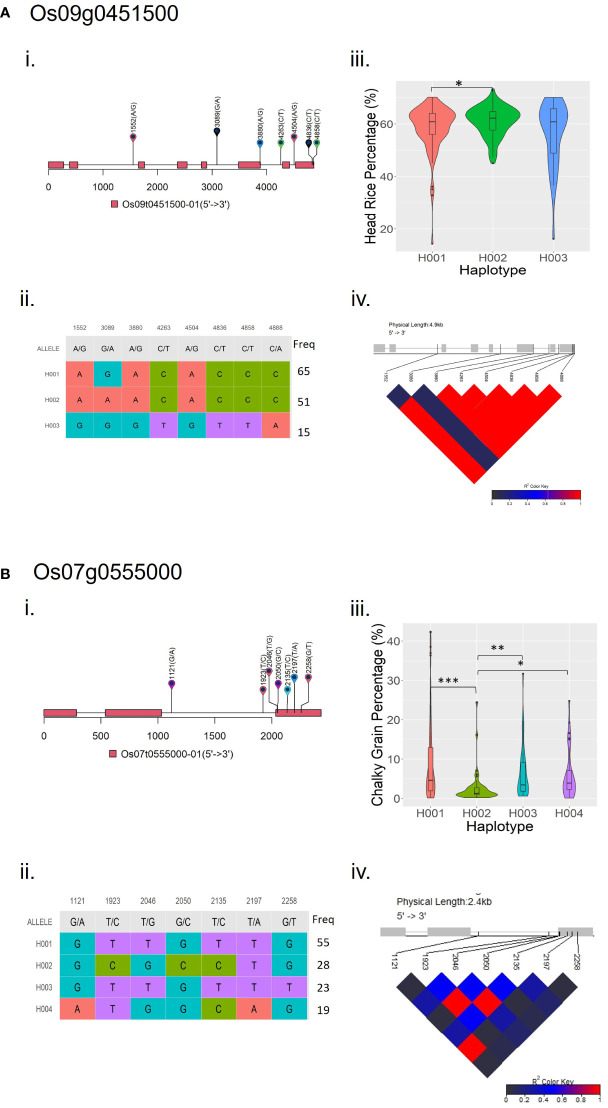
Haplotype analyses of **(A)** Os09g0451500 and **(B)** Os07g0555000, gene models associated with HRP and CGP, respectively. Information for each locus are as follows: (i) Visualization of variants position above gene model, the black line represents the genome and rectangles represent exons; (ii) Haplotype classification, each row represents a haplotype, colored columns represent loci, and the last column shows the frequency of each haplotype; (iii) Phenotype comparisons among accessions possessing different haplotypes; and (iv) LD-block visualization of each site in the locus. *Significant at *p* ≤ 0.05; **significant at *p* ≤ 0.01; ***significant at *p* ≤ 0.001.

Haplotype analyses in the Os09g0451500 and Os07g0555000, gene models associated with HRP and CGP, respectively, showed statistically significant haplotypes (**Figure 5**). In the Os09g0451500 locus, there is a significant difference (p < 0.05) between the first (H001) and second (H002) haplotypes (**Figure 5A.iii**). Rice accessions possessing haplotype H002 in the Os09g0451500 locus had the highest mean HRP at 61%. Significant differences were observed between the second (H002) and the other three haplotypes in the Os07g0555000 locus (**Figure 5B.iii**). Rice accessions possessing haplotype H002 in the Os07g0555000 locus had the lowest mean CGP at 2.7%.Three MTAs for HRP and five MTAs for CGP co-localized with previously reported QTLs (**Table 3**). For HRP, S01_14089465 is within the QTL *hr1* (Aluko et al., 2004), S07_1300806 lies within *qHR7* (Li et al., 2004), and S11_8199218 is within *qHRY11* (Zhang et al., 2020). While the top SNP in the 2018 study (S03_32703779) did not reach the significance threshold, it is located within the QTL *qHR3* (Zhang et al., 2020). Among the MTAs for CGP, S01_25791807 is within *QPGWC.NH-1.1* (Bian et al., 2014), S02_2877652 is within *qPGWC2a* (Zhao et al., 2016), S02_23451362 is within *qPGWC2c/qDEC2b* (Zhao et al., 2016), S03_35171822 is within *qPGWC3b* (Zhao et al., 2016), and S08_27534174 is within the QTL *qDEC8b* (Zhao et al., 2016).

## Discussion

### Phenotypic variability in head rice and chalky grain percentages

Phenotypic selection for high grain quality among thousands of breeding lines is laborious and time-consuming. Identification of genes responsible for head rice and chalky grain percentages using GWAS will lead to the development of functional DNA markers that will make the selection for favorable grain quality traits more efficient and effective.

Wide variations in HRP and CGP were observed among the diverse rice varieties in this study, which included *indica* and *japonica*, advanced breeding lines, and landraces. The ranges of HRP were consistent with previous studies (Zhou et al., 2015; Misra et al., 2019), but the higher tails were lower in this study (46.8% in 2018 and 42.4% in 2019 vs. 100%) due to the deliberate elimination of waxy rice accessions. HRP and CGP were inversely correlated in the BLUP, 2018, and 2019 datasets (**Figure 2**), which were consistent with similar studies (Zhou et al., 2015; Quero et al., 2018).

### Identification of marker-trait associations using genome-wide association studies and identification of candidate genes for HRP and CGP

The SNPs significantly associated with HRP and CGP varied in both years (**Table 2**). **Figure 1**, which shows the fluctuations in daily maximum, minimum and mean temperatures, rainfall, relative humidity, and solar radiation during heading to maturity (30–35 days after heading) in 2018 and 2019, suggests that the interactions between these environmental factors and genotypes significantly influenced the variation observed in HRP and CGP. Previous studies have reported the influence of genotype, environment, and genotype-by-environment effects on HRP and CGP (Blanche et al., 2009; Liu et al., 2010; Chen et al., 2013; Sreenivasulu et al., 2015; Zhou et al., 2015; Bao, 2019). Environmental factors such as temperature, rainfall, relative humidity, and nitrogen content can affect head rice and chalkiness, and the responses vary among rice genotypes (Jodari and Linscombe, 1996; Counce et al., 2005; Cooper et al., 2008; Lanning et al., 2011; Lanning and Siebenmorgen, 2013; Zhao and Fitzgerald, 2013; Rogers et al., 2016; Wada et al., 2019; Xia et al., 2021).

Among the significant MTAs detected through GWAS, significant allelic effects (p < 0.05) were found in five SNPs associated with HRP and ten SNPs associated with CGP (**Figure 4**). These SNPs will be investigated further to determine if these differences correspond to functional polymorphisms in the genes that contain these SNPs.

Nine of the significant or top MTAs reported in this study are within previously reported QTLs (S01_14089465 in *hr1* (Aluko et al., 2004), S03_32703779 in *qHR3* (Zhang et al., 2020), S07_1300806 in *qHR7* (Li et al., 2004), S11_8199218 in *qHRY11* (Zhang et al., 2020), S01_25791807 in *QPGWC.NH-1.1* (Bian et al., 2014), S02_2877652 in *qPGWC2a* (Zhao et al., 2016), S02_23451362 in *qPGWC2c/qDEC2b* (Zhao et al., 2016), S03_35171822 in *qPGWC3b* (Zhao et al., 2016), and S08_27534174 in *qDEC8b* (Zhao et al., 2016). In addition, five novel MTAs for HRP and ten novel MTAs for CGP are linked to candidate genes that are expressed in the endosperm. Some of the identified candidate genes for CGP have functions that include transcription factors and regulators, cell vesicle transport, biotic stress, protein synthesis, wall-associated kinase, cell wall modification, and development, while a few are of unknown function (Misra et al., 2019).

SNP S09_17570598, which is significantly associated with HRP, is in LD with the Os09g0451500 locus, which encodes a protein disulfide isomerase (PDI) family oxidoreductase (Kawahara et al., 2013; Sakai et al., 2013). PDI family oxidoreductases control rice grain protein composition and concentration (Onda et al., 2011; Kim et al., 2012; Onda and Kawagoe, 2013), which could affect head rice percentage (Balindong et al., 2018). S07_22868199 is significantly associated with CGP and is in LD with the Os07g0555000 locus, which encodes an F-box domain protein (Kawahara et al., 2013; Sakai et al., 2013). F-box proteins are differentially or specifically expressed during the floral transition, panicle development, and seed development stages (Jain et al., 2007). Os07g0555000 is also within the QTL *qLWR7-2*, which controls grain length-width ratio in rice (Zhong et al., 2021). Haplotype analyses in Os09g0451500 and Os07g0555000, gene models associated with HRP and CGP, respectively, showed significant differences between haplotypes with the highest HRP (H002 in **Figure 5A.iii**) and lowest CGP (H002 in **Figure 5B.iii**) with the other haplotypes in their respective loci. This information supports the results of the association analyses, and that Os09g0451500 and Os07g0555000 may be putative candidate genes for HRP and CGP, respectively.

## Conclusions

This study identified significant MTAs for CGP and HRP and reports novel genomic regions significantly associated with HRP (5 MTAs) and CGP (10 MTAs) based on the function and expression data of linked candidate genes. In addition, some of these MTAs confirmed previously reported QTLs for HRP (3 MTAs) and CGP (5 MTAs). Validation and fine mapping are necessary for the novel genomic regions found in this study.

## Data availability statement

The original contributions presented in the study are included in the article/**Supplementary Material**. Further inquiries can be directed to the corresponding authors.

## Ethics statement

The rice germplasm used in this study were obtained from the United States Department of Agriculture (USDA) National Plant Germplasm System (NPGS) and from the inbred and specialty rice breeding programs of the Texas A&M AgriLife Research at Beaumont. The collection and use of rice germplasm, as well as the methods conducted in this study complies with relevant institutional, national, and international guidelines and legislation.

## Author contributions

DS: Conceptualization, Data curation, Formal Analysis, Investigation, Methodology, Software, Supervision, Visualization, Writing – original draft. SS: Conceptualization, Funding acquisition, Investigation, Methodology, Project administration, Resources, Supervision, Writing – review & editing. LW: Funding acquisition, Writing – review & editing.
