# Supplementary material for: Genetic architecture of head rice and rice chalky grain percentages using genome-wide association studies
Source: Front Plant Sci. 2023 Nov 17;14:1274823. doi: 10.3389/fpls.2023.1274823 (PMC10691675; doi:10.3389/fpls.2023.1274823)
Supplement: Supplementary file 1 [file DataSheet_1.pdf]

Supplementary Material

Table S1. Head rice and chalky grain percentages of diverse rice accessions grown in Texas A&M AgriLife Research at Beaumont in 2018 and 2019.

	ID	Head Rice Percentage (%)		Chalky Grain Percentage (%)	
		2018	2019	2018	2019
1	119A/170R	49.1	48.2	19.4	15.9
2	119A/173R	52.1	60.8	9.9	5.3
3	119B	60.5	63.7	15.4	3.4
4	152R	50.6	55.0	11.5	2.6
5	153R	45.0	36.5	13.4	5.0
6	163R	43.3	46.0	4.4	2.5
7	164R	-	48.9	-	1.6
8	170R	56.2	47.3	4.8	1.3
9	171R	55.0	48.8	2.1	1.3
10	172R	39.2	43.8	12.0	19.4
11	173R	59.1	56.5	1.0	2.7
12	329A/170R	55.1	47.0	9.8	6.2
13	329A/173R	53.3	65.3	8.0	4.4
14	329B	-	67.5	-	3.4
15	339A/170R	44.7	39.9	21.7	7.4
16	339A/173R	50.4	62.2	7.3	4.1
17	339B	60.0	35.1	13.6	5.9
18	435A/170R	-	61.9	-	10.1
19	435A/173R	49.9	61.7	8.3	3.0
20	435B	56.7	54.8	30.0	9.0
21	56-122-23	62.0	62.7	21.0	7.0
22	89-Y-235	57.0	65.8	15.0	2.2
23	A 152	56.2	59.8	3.6	0.5
24	Agostano	49.9	62.6	35.6	20.4

	ID	Head Rice Percentage (%)		Chalky Grain Percentage (%)	
		2018	2019	2018	2019
25	Ai Yeh Lu	55.4	62.8	25.4	12.9
26	Antonio	63.7	57.6	4.0	3.4
27	Ardito	42.6	62.5	39.7	11.1
28	B6616A4-22-Bk-5-4	61.0	69.0	0.7	0.2
29	Baber	61.5	59.5	36.9	42.4
30	Bamoa A75 (LG)	55.3	63.3	42.9	24.5
31	Bhim Dhan	50.0	65.2	31.5	27.8
32	Biser 1	50.0	64.4	33.1	18.0
33	Blue Rose Supreme	50.7	60.9	3.2	2.1
34	Blue Stick	61.7	50.7	-	16.1
35	Bombilla	66.0	66.2	15.5	7.4
36	Bombon	57.1	14.3	41.7	12.7
37	Botika S/R	52.3	60.6	17.3	19.3
38	British Honduras Creole	41.1	60.9	4.2	2.0
39	Bul Zo	59.1	53.9	12.8	4.3
40	Buphopa	63.2	50.5	4.8	9.9
41	C 5560	36.1	-	-	-
42	C 8429	52.6	49.6	31.0	2.9
43	C4-63	60.1	61.4	0.3	0.6
44	C57-5043	53.2	-	5.4	-
45	Calmochi-101	55.1	67.9	-	-
46	Camponi SML	36.9	63.2	8.7	2.3
47	Celiaj	64.4	57.7	9.9	23.7
48	Cenit	44.5	67.9	-	0.6
49	Chacareiro Uruguay	59.9	65.9	40.8	22.0
50	Cheniere	64.0	57.6	1.6	1.0
51	Chia Nung Yu 242	45.5	5.9	5.7	24.8
52	Chunjiangzao No. 1	66.5	65.9	40.7	8.9
53	Cocodrie	64.6	62.0	3.5	2.3
54	Colorado	52.9	57.1	4.5	0.9
55	Coppocina	45.0	63.4	14.8	15.7

	ID	Head Rice Percentage (%)		Chalky Grain Percentage (%)	
		2018	2019	2018	2019
56	Criollo Chivacoa 2	54.7	45.0	4.2	1.4
57	Csornuj	56.7	60.6	17.3	37.0
58	Cybonnet	62.9	58.9	1.9	0.3
59	Cypress	65.3	63.1	1.4	0.9
60	Darmali	52.9	-	-	-
61	Della 2	55.7	60.8	12.7	2.9
62	Diamond	38.0	56.8	8.8	3.3
63	Early	36.4	47.4	41.5	16.2
64	Early Colusa	61.4	64.0	38.5	36.4
65	Early Wataribune	63.8	45.5	5.2	22.1
66	Edith	50.5	60.1	10.1	2.7
67	Edomen Scented	60.7	65.7	6.3	1.1
68	Egyptian Wild Type	58.7	-	8.8	-
69	El Paso L-144	62.7	66.6	7.1	1.3
70	Erythroceros Hokkaido	65.2	56.6	6.4	2.8
71	Ginmasari	54.2	57.8	11.0	8.0
72	Gogo Lempuk	59.6	63.3	22.9	33.4
73	GPNO 1106	47.8	64.5	6.8	4.3
74	Guatemala 1021	54.4	62.8	34.9	3.3
75	H57-3-1	45.5	66.6	18.9	5.8
76	Habiganj Boro 6	63.9	60.9	29.0	42.3
77	Haginomae Mochi	59.7	56.1	-	-
78	Hatsunishiki	63.2	65.9	23.2	8.9
79	HB-6-2	56.0	50.0	21.6	17.1
80	IAC 25	48.9	66.3	15.9	6.8
81	IR 1321-12	61.9	48.5	0.1	0.1
82	IR24	58.8	64.6	-	0.5
83	IR-44595	43.4	72.3	-	4.7
84	IR64	57.4	49.3	13.9	15.0
85	IR64-Sub1	60.4	61.3	2.9	2.4
86	IR72	58.5	62.4	1.2	18.5

	ID	Head Rice Percentage (%)		Chalky Grain Percentage (%)	
		2018	2019	2018	2019
87	IRAT 177	60.5	64.9	4.0	4.8
88	IRAT 44	55.7	67.0	24.6	5.5
89	Italica Carolina	56.9	57.1	9.8	4.6
90	J.P. 5	62.5	56.0	4.9	3.0
91	Jefferson	62.9	69.7	3.4	1.0
92	Jouiku 393G	64.1	62.8	3.3	2.4
93	Kamenoo	66.6	69.2	11.4	0.9
94	Karabaschak	59.3	-	17.2	-
95	Karang Serang	50.0	59.6	10.7	13.7
96	Katy	62.5	59.7	0.4	1.0
97	Kaukkyi Ani	53.1	36.1	-	14.6
98	Kaybonnet	65.8	65.8	0.3	0.2
99	Khao Luang	45.3	-	-	-
100	Khao Phoi	30.7	36.7	-	-
101	Kiuki No. 46	60.3	56.8	4.3	1.3
102	Koshihikari	63.1	65.8	8.4	0.5
103	KRASNODARSKIJ 3352	64.5	64.6	13.4	9.4
104	KRASNODARSKIJ 424	62.2	63.5	10.6	9.1
105	L-201	39.0	67.6	14.9	2.1
106	L-202	55.0	60.0	9.4	0.7
107	L-203	39.3	49.3	11.2	2.7
108	LAC 23	46.3	64.8	7.8	9.3
109	Lacassine	58.6	66.3	8.9	1.8
110	Lady Wright Seln	56.8	70.1	19.9	4.0
111	LaGrue	46.4	57.9	19.0	3.5
112	LaKast	44.8	63.9	16.6	3.2
113	Leah	48.7	64.6	18.1	2.5
114	Lebonnet	58.5	60.9	1.0	0.2
115	Lemont	51.9	67.8	11.8	1.0
116	LGRU 2	48.5	-	10.9	-
117	Ligerito	53.9	61.1	26.6	4.1

ID		Head Rice Percentage (%)		Chalky Grain Percentage (%)	
		2018	2019	2018	2019
118	Lua Chua Chan	54.6	-	-	-
119	Luk Takhar	62.5	68.9	7.2	13.0
120	Lusitano	62.1	53.9	31.8	6.8
121	M-201	67.1	57.5	2.9	3.4
122	M-202	65.6	54.2	6.1	1.9
123	M-203	61.7	70.2	5.5	1.9
124	M-204	64.5	63.6	1.7	0.5
125	M-401	63.8	45.1	1.9	1.7
126	Mars	64.1	63.8	0.5	1.0
127	Mercury	66.7	67.3	1.0	0.9
128	Minghui 63	56.3	58.5	2.1	3.4
129	Mojito Colorado	59.7	60.2	34.9	16.6
130	Moroberekan	58.9	-	12.7	-
131	N22	67.6	69.2	12.7	1.1
132	Nanton No. 131	64.5	55.6	-	38.6
133	Newbonnet	60.8	55.0	8.7	1.1
134	Nipponbare	64.9	62.6	4.9	1.7
135	Niwahutaw Mochi	63.1	53.7	-	-
136	Nortai	64.2	64.6	11.5	7.1
137	Nova	59.1	59.7	1.5	0.6
138	Noventa Dias Blanco	65.3	63.4	27.0	10.3
139	NSF-TV 107	53.3	68.1	14.6	5.8
140	Oryzica Llanos 4	62.9	54.0	0.3	0.8
141	Oryzica Llanos 5	46.2	58.7	-	3.8
142	OS 6 (WC 10296)	44.1	62.1	25.2	4.1
143	Padi Pohon Batu	42.9	32.8	18.9	11.3
144	Palmyra	62.0	66.3	0.0	0.1
145	Panda	39.5	66.4	10.9	2.4
146	Pergonil 15	63.7	64.4	45.0	15.9
147	Presidio	62.3	58.8	0.7	0.7
148	Quinimpol	60.8	53.3	-	-

		Head Rice Percentage (%)		Chalky Grain Percentage (%)	
	ID	2018	2019	2018	2019
149	R 101	52.8	61.9	17.2	4.6
150	R 67	35.3	16.2	21.2	5.9
151	R 75	48.1	63.3	23.4	9.5
152	Ragasu	60.4	59.1	14.8	1.9
153	RD 218	57.4	57.0	-	2.3
154	Rex	55.3	63.2	7.8	0.5
155	Rico 1	62.6	64.2	0.7	1.8
156	Romanica	42.7	56.4	21.1	3.5
157	Romeno	62.3	-	10.2	-
158	Romeo	48.9	59.0	38.6	6.1
159	Rondo	57.8	62.3	8.9	4.1
160	ROY J	54.9	59.3	2.7	2.4
161	RT 1031-69	53.2	63.1	2.4	4.4
162	RU-1003098	64.6	60.8	5.3	2.6
163	RU-1003123	63.2	61.3	7.9	1.2
164	RU-1303181	62.9	60.3	1.4	1.6
165	RU-1303184	66.6	64.1	1.6	1.1
166	RU-1403138	64.6	63.5	6.1	1.3
167	RU-1403141	66.2	63.7	0.9	0.4
168	RU-1403166	65.0	66.8	1.3	0.8
169	RU-1503110	68.3	57.2	0.6	0.2
170	RU-1503147	57.6	64.5	3.8	2.7
171	RU-1603126	58.4	73.4	1.5	0.7
172	RU-1603150	59.1	-	1.4	-
173	RU-903147	64.0	66.6	5.9	1.1
174	Saber	65.9	55.8	0.3	6.1
175	Sabine	65.6	61.3	2.6	3.5
176	Sathi	59.0	66.9	1.8	1.1
177	Saturn	59.0	62.4	0.8	1.6
178	Secano do Brazil	60.1	65.8	2.4	6.5
179	Sel. No. 388	60.2	55.8	10.1	19.7

ID		Head Rice Percentage (%)		Chalky Grain Percentage (%)	
		2018	2019	2018	2019
180	Shimizu Mochi	60.6	63.8	-	-
181	Shinriki	59.1	55.3	18.5	15.7
182	Shirogane	66.1	61.4	6.8	1.6
183	Short Grain	65.1	50.3	-	8.8
184	Simpor	63.1	56.0	0.2	6.8
185	Sipirasikkam	56.4	60.0	23.2	12.9
186	Sml Kapuri	59.1	62.5	13.6	3.6
187	Somewake	58.6	66.2	4.2	5.7
188	Sri Malaysia Dua	63.2	64.8	0.4	2.3
189	Sultani	66.9	61.7	0.3	1.4
190	Sung Liao 2	66.6	48.4	3.0	1.2
191	Suweon	61.2	61.9	11.3	5.1
192	Ta Hung Ku	57.3	63.3	22.6	4.1
193	Taichu Mochi 59	68.6	62.9	1.5	1.5
194	Tainan-Iku No. 512	61.4	57.3	6.5	4.9
195	Taipei 309	62.0	61.1	4.7	6.7
196	Takao No. 25	62.2	63.5	11.5	4.6
197	Takao-Iku No. 44	62.8	61.7	15.8	1.3
198	Takao-Iku No. 8	65.5	62.2	6.6	5.1
199	Tamanishiki	59.4	50.4	8.8	2.1
200	Terso	58.8	55.0	2.1	1.3
201	Thad	60.3	59.2	4.3	1.8
202	Tia Bura	48.8	60.1	0.6	11.8
203	Titan	65.9	69.0	7.5	1.1
204	Tokyo Shino Mochi	57.5	64.1	-	-
205	Tox 782-20-1	51.2	53.6	17.5	0.7
206	Upland	38.3	66.5	34.3	2.8
207	UZ ROSZ M38	45.6	53.1	9.3	31.7
208	Vary Tarva Osla	55.6	65.9	7.0	2.3
209	Very Early M9	61.7	64.3	23.0	2.1
210	WAB462-10-3-1	60.6	65.5	2.4	1.5

	ID	Head Rice Percentage (%)		Chalky Grain Percentage (%)	
		2018	2019	2018	2019
211	WC 2811	46.6	65.7	16.7	2.5
212	WC 3397	60.8	66.2	21.4	1.6
213	WC 3532	48.4	-	-	-
214	WC 4443	46.4	59.1	34.4	28.3
215	WC 6	49.3	58.4	-	-
216	Wells	49.6	60.3	5.7	0.7
217	WIR 3039	63.1	-	46.8	-
218	WW 8/2290	43.5	52.0	6.2	5.7
219	Yong Chal Byo	53.6	65.2	9.0	2.8
220	Zhenshan 2	34.9	66.9	39.6	15.3

Table S2. List of gene models located within or in linkage disequilibrium to SNPs significantly associated with head rice percentage (HRP) and chalky grain percentage (CGP) in rice.

SNP	Locus (IRGSP Build 5)	Chromosome	Location (IRGSP Build 5)		DNA Strand Direction	Gene Product	Annotation
			Start (bp)	End (bp)			
**Trait: Head Rice Percentage**							
S01_14089465	Os01g0347800	1	13990641	13993351	-	Os01t0347800-00	Hypothetical conserved gene
	Os01g0348000	1	14002135	14002430	+	Os01t0348000-00	Similar to Papain-like cysteine proteinase (Fragment)
	Os01g0348600	1	14029951	14035863	+	Os01t0348600-01	Similar to MFP2 (Fatty acid multifunctional protein) (AtMFP2)
						Os01t0348600-02	Similar to glyoxysomal fatty acid beta-oxidation multifunctional protein MFP-a
						Os01t0348600-03	Similar to glyoxysomal fatty acid beta-oxidation multifunctional protein MFP-a
	Os01g0348700	1	14038978	14040799	+	Os01t0348700-01	Similar to 60S ribosomal protein L23a (L25)
	Os01g0348800	1	14042202	14043408	-	Os01t0348800-01	Similar to Salt-stress induced protein (Salt protein)
	Os01g0348900	1	14045964	14047305	-	Os01t0348900-01	Jacalin-related mannose-binding lectin, Salinity tolerance, Resistance to M. oryzae
						Os01t0348900-02	SalT gene product (Salt-induced protein)
	Os01g0349000	1	14053913	14055501	-	Os01t0349000-01	Conserved hypothetical protein
	Os01g0349400	1	14065180	14070132	-	Os01t0349400-01	Similar to Serine/threonine protein phosphatase PP1 (EC 3.1.3.16) (Fragment)
	Os01g0349600	1	14077175	14078533	+	Os01t0349600-01	Conserved hypothetical protein
	Os01g0349800	1	14083997	14087284	+	Os01t0349800-01	Similar to Cytochrome P450
	Os01g0350000	1	14100193	14101903	+	Os01t0350000-01	Transferase family protein
	Os01g0350101	1	14104086	14105085	-	Os01t0350101-00	Hypothetical gene
	Os01g0350200	1	14120643	14122217	+	Os01t0350200-00	Similar to P450
	Os01g0350500	1	14129174	14131419	-	Os01t0350500-01	Conserved hypothetical protein

SNP	Locus (IRGSP Build 5)	Chromosome	Location (IRGSP Build 5)		DNA Strand Direction	Gene Product	Annotation
			Start (bp)	End (bp)			
	Os01g0350900	1	14155196	14162115	+	Os01t0350900-01	Similar to VIP2 protein
						Os01t0350900-02	RING-finger E3 ligase, Regulation of plant architecture
	Os01g0351100	1	14177770	14183487	+	Os01t0351100-00	Similar to Poly
	Os02g0278400	2	10290998	10297670	+	Os02t0278400-01	Cytochrome P450 family protein
						Os02t0278400-02	Similar to Cytochrome P450 monooxygenase CYP71U4v2
	Os02g0278700	2	10302717	10312725	-	Os02t0278700-01	Similar to Kaurene synthase A (Fragment)
	Os02g0279400	2	10348276	10349009	-	Os02t0279400-00	Hypothetical conserved gene
	Os02g0279600	2	10359697	10362783	+	Os02t0279600-00	Similar to TOM2B
	Os02g0279800	2	10367760	10371348	+	Os02t0279800-01	Conserved hypothetical protein
	Os02g0279850	2	10373195	10373635	-	Os02t0279850-00	Hypothetical genes
	Os02g0280000	2	10380704	10381358	-	Os02t0280000-01	Hypothetical gene
	Os02g0280100	2	10381383	10383070	-	Os02t0280100-00	Micro-fibrillar-associated 1, C-terminal family protein
S02_10354220	Os02g0280200	2	10388492	10390067	+	Os02t0280200-00	Similar to Xet3 protein
	Os02g0280300	2	10392976	10394548	+	Os02t0280300-00	Similar to Xyloglucan endotransglycosylase (Fragment)
	Os02g0280400	2	10400072	10409937	+	Os02t0280400-01	Similar to casein kinase I isoform delta-like
						Os02t0280400-02	Similar to Dual specificity kinase 1
						Os02t0280400-03	Hypothetical gene
	Os02g0280500	2	10407455	10408195	-	Os02t0280500-01	Similar to Glyoxalase I
	Os02g0280700	2	10415297	10419287	+	Os02t0280700-01	Similar to Iron/ascorbate-dependent oxidoreductase
	Os02g0281000	2	10448060	10457012	+	Os02t0281000-01	RmlC-like jelly roll fold domain containing protein
						Os02t0281000-02	Similar to predicted protein
S06_14934656	Os06g0358800	6	14838313	14841945	+	Os06t0358800-01	Ribonuclease III domain containing protein

SNP	Locus (IRGSP Build 5)	Chromosome	Location (IRGSP Build 5)		DNA Strand Direction	Gene Product	Annotation
			Start (bp)	End (bp)			
	Os06g0359400	6	14877951	14883774	-	Os06t0359400-01	Conserved hypothetical protein
	Os06g0360300	6	14948278	14960857	-	Os06t0360300-01	NAD(P)-binding domain containing protein
	Os06g0360500	6	14960182	14961613	+	Os06t0360500-01	Conserved hypothetical protein
	Os06g0360600	6	14963814	14981392	-	Os06t0360600-01	Hypothetical protein
						Os06t0360600-02	Hypothetical conserved gene
						Os06t0360600-03	Hypothetical conserved gene
	Os06g0361500	6	15020666	15026987	-	Os06t0361500-01	Similar to cDNA clone:J013000C15, full insert sequence
						Os06t0361500-02	Conserved hypothetical protein
	Os07g0123400	7	1201315	1202025	-	Os07t0123400-01	Conserved hypothetical protein
	Os07g0123450	7	1201528	1202538	+	Os07t0123450-01	Conserved hypothetical protein
	Os07g0123500	7	1212610	1216544	-	Os07t0123500-01	BTB/POZ fold domain containing protein
	Os07g0123601	7	1227519	1237226	+	Os07t0123601-01	Hypothetical gene
	Os07g0123700	7	1227856	1237253	-	Os07t0123700-00	PUL domain containing protein
	Os07g0123800	7	1238804	1239626	-	Os07t0123800-01	Uncharacterised conserved protein UCP031279 domain containing protein
	Os07g0123900	7	1244458	1245177	-	Os07t0123900-01	Conserved hypothetical protein
	Os07g0124000	7	1247874	1249251	+	Os07t0124000-01	Conserved hypothetical protein
S07_1300806	Os07g0124100	7	1254047	1254744	-	Os07t0124100-01	Similar to Phytosulfokines 4 precursor
	Os07g0124300	7	1266873	1267776	+	Os07t0124300-00	Similar to bZIP transcription factor family protein
	Os07g0124400	7	1268041	1268855	+	Os07t0124400-01	Hypothetical conserved gene
	Os07g0124500	7	1273555	1277717	+	Os07t0124500-01	Similar to Eukaryotic translation initiation factor 3 subunit 8 (eIF3 p110) (eIF3c)
	Os07g0124600	7	1280407	1284040	+	Os07t0124600-01	Nucleotide-binding, alpha-beta plait domain containing protein
	Os07g0124650	7	1297865	1298428	-	Os07t0124650-00	Hypothetical gene
	Os07g0124700	7	1298598	1303299	+	Os07t0124700-01	Similar to PLETHORA1

SNP	Locus (IRGSP Build 5)	Chromosome	Location (IRGSP Build 5)		DNA Strand Direction	Gene Product	Annotation
			Start (bp)	End (bp)			
	Os07g0124750	7	1304291	1307060	-	Os07t0124750-00	Similar to cDNA clone:J033097L05, full insert sequence
	Os07g0124800	7	1312767	1315444	-	Os07t0124800-01	Similar to Chaperone protein dnaJ
	Os07g0124900	7	1318034	1318789	+	Os07t0124900-01	Allergen V5/Tpx-1 related family protein
	Os07g0125000	7	1321193	1322127	-	Os07t0125000-01	Similar to Pathogenesis-related protein PR-1 precursor
						Os07t0125000-02	Similar to Pathogenesis-related protein PR-1 precursor
	Os07g0125201	7	1334405	1334923	+	Os07t0125201-00	Allergen V5/Tpx-1 related family protein
	Os07g0125500	7	1351187	1351942	+	Os07t0125500-01	Allergen V5/Tpx-1 related family protein
	Os07g0125600	7	1354346	1355280	-	Os07t0125600-01	Similar to Pathogenesis-related protein PR-1 precursor
						Os07t0125600-02	Similar to Pathogenesis-related protein PR-1 precursor
	Os07g0126100	7	1367558	1368076	+	Os07t0126100-00	Allergen V5/Tpx-1 related family protein
	Os07g0126301	7	1384340	1385095	+	Os07t0126301-01	Allergen V5/Tpx-1 related family protein
	Os07g0126401	7	1387499	1388433	-	Os07t0126401-01	Similar to Pathogenesis-related protein PR-1 precursor
						Os07t0126401-02	Similar to Pathogenesis-related protein PR-1 precursor
	Os07g0292100	7	11369736	11373215	-	Os07t0292100-00	Hypothetical conserved gene
	Os07g0292800	7	11391450	11394315	+	Os07t0292800-01	Ribosomal protein L24e domain containing protein
	Os07g0293000	7	11415241	11420370	-	Os07t0293000-00	Hypothetical conserved gene
S07_11468500	Os07g0294300	7	11483278	11483767	+	Os07t0294300-01	Hypothetical conserved gene
	Os07g0294600	7	11513400	11514053	+	Os07t0294600-01	Protein prenyltransferase domain containing protein
	Os07g0294650	7	11517056	11517624	+	Os07t0294650-01	Non-protein coding transcript
	Os07g0294700	7	11517972	11519318	-	Os07t0294700-01	Conserved hypothetical protein

SNP	Locus (IRGSP Build 5)	Chromosome	Location (IRGSP Build 5)		DNA Strand Direction	Gene Product	Annotation
			Start (bp)	End (bp)			
S08_18460399	Os07g0294800	7	11529039	11537292	+	Os07t0294800-01	Conserved hypothetical protein
	Os07g0295000	7	11537937	11541785	-	Os07t0295000-01	Mitochondrial carrier protein domain containing protein
						Os07t0295000-02	Mitochondrial carrier protein domain containing protein
	Os07g0295200	7	11546191	11550286	-	Os07t0295200-01	Protein of unknown function DUF167 family protein
	Os07g0295400	7	11560158	11565849	+	Os07t0295400-01	Conserved hypothetical protein
						Os07t0295400-02	Conserved hypothetical protein
	Os07g0295601	7	11567276	11576881	+	Os07t0295601-00	Hypothetical conserved gene
	Os08g0387050	8	18365587	18368454	-	Os08t0387050-01	Hypothetical conserved gene
	Os08g0387200	8	18379880	18387142	+	Os08t0387200-01	Protein of unknown function DUF81 family protein
	Os08g0387400	8	18393372	18397369	+	Os08t0387400-01	Similar to Cellulase (Fragment)
	Os08g0387500	8	18397603	18400148	-	Os08t0387500-01	Similar to Sulfated surface glycoprotein 185 precursor (SSG 185)
	Os08g0387700	8	18407104	18417919	-	Os08t0387700-01	NB-ARC domain containing protein
						Os08t0387700-02	Hypothetical conserved gene
						Os08t0387700-03	Hypothetical conserved gene
	Os08g0388300	8	18433871	18447043	+	Os08t0388300-01	NB-ARC domain containing protein
						Os08t0388300-02	Hypothetical conserved gene
	Os08g0388900	8	18459714	18467509	+	Os08t0388900-00	Similar to para-hydroxybenzoate--polyprenyltransferase
	Os08g0389300	8	18494243	18495717	+	Os08t0389300-00	Similar to para-hydroxybenzoate--polyprenyltransferase
	Os08g0389500	8	18502775	18507603	-	Os08t0389500-01	Integrase, catalytic core domain containing protein
	Os08g0389601	8	18537772	18538285	-	Os08t0389601-01	Non-protein coding transcript
	Os08g0389700	8	18540704	18546061	+	Os08t0389700-01	Hypothetical conserved gene

SNP	Locus (IRGSP Build 5)	Chromosome	Location (IRGSP Build 5)		DNA Strand Direction	Gene Product	Annotation
			Start (bp)	End (bp)			
						Os08t0389700-02	Protein of unknown function DUF81 family protein
						Os08t0389700-03	Similar to cDNA clone:001-103-F02, full insert sequence
	Os08g0389733	8	18548980	18550312	-	Os08t0389733-01	Hypothetical gene
	Os08g0389901	8	18553972	18555260	-	Os08t0389901-00	Non-protein coding transcript
S09_17570598	Os09g0448500	9	17471795	17473819	+	Os09t0448500-00	Hypothetical conserved gene
	Os09g0448900	9	17487074	17490814	+	Os09t0448900-01	Conserved hypothetical protein
	Os09g0449000	9	17493924	17494657	+	Os09t0449000-01	Hypothetical conserved gene
	Os09g0449400	9	17528424	17531382	+	Os09t0449400-01	Hypothetical conserved gene
	Os09g0449500	9	17534921	17536472	+	Os09t0449500-01	Conserved hypothetical protein
	Os09g0449600	9	17540550	17544597	+	Os09t0449600-01	Similar to Aconitate hydratase, cytoplasmic (EC 4.2.1.3) (Citrate hydro-lyase) (Aconitase)
						Os09t0449600-02	Similar to F-box protein interaction domain containing protein, expressed
	Os09g0449650	9	17546212	17546955	-	Os09t0449650-00	Conserved hypothetical protein
	Os09g0449700	9	17548321	17549952	+	Os09t0449700-01	Hypothetical gene
	Os09g0449800	9	17553789	17554631	+	Os09t0449800-01	Conserved hypothetical protein
	Os09g0450200	9	17558287	17560657	+	Os09t0450200-01	Conserved hypothetical protein
	Os09g0450300	9	17561352	17567063	-	Os09t0450300-01	MAP65/ASE1 family protein
						Os09t0450300-02	MAP65/ASE1 family protein
						Os09t0450300-03	Microtubule-associated protein MAP65-1a
	Os09g0450600	9	17581033	17582023	+	Os09t0450600-00	Similar to Protein HVA22
	Os09g0450700	9	17585406	17586408	-	Os09t0450700-01	Conserved hypothetical protein
	Os09g0451000	9	17592043	17593604	-	Os09t0451000-01	Similar to 1-aminocyclopropane-1-carboxylate oxidase 1 (EC 1.14.17.4) (ACC oxidase 1) (Ethylene-forming enzyme) (EFE)

SNP	Locus (IRGSP Build 5)	Chromosome	Location (IRGSP Build 5)		DNA Strand Direction	Gene Product	Annotation
			Start (bp)	End (bp)			
						Os09t0451000-02	Similar to 1-aminocyclopropane-1-carboxylate oxidase 1 (EC 1.14.17.4) (ACC oxidase 1) (Ethylene-forming enzyme) (EFE)
	Os09g0451133	9	17592439	17593552	+	Os09t0451133-00	Hypothetical gene
	Os09g0451266	9	17631419	17632730	-	Os09t0451266-00	Hypothetical gene
	Os09g0451400	9	17631456	17633045	+	Os09t0451400-01	1-aminocyclopropane-1-carboxylate oxidase 1 (EC 1.14.17.4) (ACC oxidase 1) (Ethylene-forming enzyme) (EFE)
	Os09g0451500	9	17636986	17641903	+	Os09t0451500-01	Similar to protein disulfide isomerase
						Os09t0451500-02	Thioredoxin domain 2 containing protein
	Os09g0451700	9	17647754	17649397	+	Os09t0451700-00	Protein of unknown function DUF573 family protein
	Os09g0451800	9	17651130	17653983	-	Os09t0451800-01	Protein of unknown function DUF1644 family protein
	Os09g0452200	9	17664543	17668139	+	Os09t0452200-01	Similar to LysM-domain GPI-anchored protein 1 precursor.Splice isoform 2
						Os09t0452200-02	Similar to LysM-domain GPI-anchored protein 1 precursorSplice isoform 2
	Os09g0452300	9	17669275	17671799	+	Os09t0452300-01	Similar to cDNA, clone: J065054A13, full insert sequence
	Os11g0249900	11	8097927	8100411	+	Os11t0249900-01	Protein kinase-like domain containing protein
						Os11t0249900-02	Similar to Protein kinase domain containing protein, expressed
S11_8199218	Os11g0250000	11	8101612	8105927	-	Os11t0250000-01	Similar to cDNA clone:J013065B13, full insert sequence
	Os11g0250100	11	8106731	8108209	+	Os11t0250100-01	Conserved hypothetical protein
	Os11g0250400	11	8129467	8137748	-	Os11t0250400-01	Ankyrin repeat containing protein
	Os11g0251250	11	8159671	8160298	-	Os11t0251250-01	Hypothetical protein
	Os11g0251400	11	8164224	8170982	-	Os11t0251400-01	Hypothetical conserved gene

SNP	Locus (IRGSP Build 5)	Chromosome	Location (IRGSP Build 5)		DNA Strand Direction	Gene Product	Annotation
			Start (bp)	End (bp)			
						Os11t0251400-02	Ankyrin repeat containing protein
						Os11t0251400-03	Hypothetical conserved gene
	Os11g0252400	11	8199192	8202833	-	Os11t0252400-01	Ankyrin repeat containing protein
						Os11t0252400-02	Hypothetical conserved gene
						Os11t0252400-03	Hypothetical conserved gene
	Os11g0252900	11	8224737	8226181	+	Os11t0252900-00	Similar to retrotransposon protein
**Trait: Chalky Grain Percentage**							
S01_25791807	Os01g0609200	1	25695656	25704307	-	Os01t0609200-00	Similar to Pleiotropic drug resistance protein 3
	Os01g0609300	1	25705751	25712070	-	Os01t0609300-01	Similar to Pleiotropic drug resistance protein 3
						Os01t0609300-02	Hypothetical conserved gene
	Os01g0609401	1	25709090	25711426	+	Os01t0609401-00	Hypothetical gene
	Os01g0609700	1	25733423	25735102	+	Os01t0609700-01	Conserved hypothetical protein
	Os01g0609900	1	25737767	25745968	-	Os01t0609900-01	Similar to Pleiotropic drug resistance protein 4
						Os01t0609900-02	Similar to PDR-like ABC transporter (PDR4 ABC transporter)
	Os01g0609950	1	25740834	25743695	+	Os01t0609950-00	Hypothetical gene
	Os01g0610050	1	25755592	25756158	-	Os01t0610050-00	Non-protein coding transcript
	Os01g0610100	1	25757306	25760302	+	Os01t0610100-01	Similar to Clone ZZZ51 mRNA sequence
	Os01g0610300	1	25767552	25774973	+	Os01t0610300-01	Bromo adjacent region domain containing protein
	Os01g0610400	1	25776572	25779527	+	Os01t0610400-01	Acyl-CoA N-acyltransferase domain containing protein
	Os01g0610500	1	25781721	25784770	-	Os01t0610500-01	Yip1 domain containing protein
						Os01t0610500-02	Similar to Yip1 domain family member 1

SNP	Locus (IRGSP Build 5)	Chromosome	Location (IRGSP Build 5)		DNA Strand Direction	Gene Product	Annotation
			Start (bp)	End (bp)			
	Os01g0610600	1	25786330	25793253	-	Os01t0610600-01	Endonuclease/exonuclease/phosphatase domain containing protein
						Os01t0610600-02	Hypothetical conserved gene
	Os01g0610700	1	25794032	25796876	-	Os01t0610700-01	Zinc finger, RING/FYVE/PHD-type domain containing protein
	Os01g0610800	1	25798926	25800900	-	Os01t0610800-01	Thrombospondin, type I repeat containing protein
	Os01g0611000	1	25811792	25814660	+	Os01t0611000-01	Similar to Unidentified precursor
						Os01t0611000-02	Hypothetical conserved gene
	Os01g0611100	1	25817993	25820475	+	Os01t0611100-01	Similar to GTP-binding nuclear protein Ran-2
	Os01g0611300	1	25826185	25827297	+	Os01t0611300-01	Conserved hypothetical protein
	Os01g0611900	1	25870938	25875911	-	Os01t0611900-01	Pentatricopeptide repeat domain containing protein
						Os01t0611900-02	Pentatricopeptide repeat domain containing protein
	Os01g0611950	1	25875220	25875972	+	Os01t0611950-00	Hypothetical gene
	Os01g0612000	1	25876550	25880042	-	Os01t0612000-01	Similar to DNA methyltransferase PMT1-like protein
	Os01g0612100	1	25880873	25881592	+	Os01t0612100-00	Similar to ribosomal protein S11 containing protein
	Os01g0612200	1	25886060	25890621	+	Os01t0612200-01	Cytochrome c oxidase, subunit Vb family protein
S01_36699691	Os01g0818600	1	36598094	36602166	-	Os01t0818600-01	Serine/threonine protein kinase-related domain containing protein
						Os01t0818600-02	Similar to cDNA clone:J013021N20, full insert sequence
	Os01g0818700	1	36602743	36610319	-	Os01t0818700-01	Leucine-rich repeat, N-terminal domain containing protein
	Os01g0818800	1	36612697	36614114	-	Os01t0818800-01	Hypothetical protein
	Os01g0818900	1	36615030	36618374	-	Os01t0818900-01	Conserved hypothetical protein

SNP	Locus (IRGSP Build 5)	Chromosome	Location (IRGSP Build 5)		DNA Strand Direction	Gene Product	Annotation
			Start (bp)	End (bp)			
	Os01g0819000	1	36617995	36622334	+	Os01t0819000-01	Similar to transposon protein CACTA, En/Spm sub-class
						Os01t0819000-02	Conserved hypothetical protein
	Os01g0819100	1	36629670	36631395	+	Os01t0819100-01	Protein kinase-like domain containing protein
	Os01g0819200	1	36631794	36636657	-	Os01t0819200-00	Similar to calcium lipid binding protein-like
	Os01g0819233	1	36634011	36638199	+	Os01t0819233-01	Hypothetical gene
	Os01g0819266	1	36634447	36634980	-	Os01t0819266-00	Non-protein coding transcript
	Os01g0819300	1	36641248	36643435	-	Os01t0819300-01	Conserved hypothetical protein
	Os01g0819400	1	36646198	36649319	+	Os01t0819400-00	Similar to Ubiquitin carrier protein
	Os01g0819433	1	36647361	36649092	-	Os01t0819433-00	Non-protein coding gene
	Os01g0819466	1	36668708	36671292	-	Os01t0819466-00	Non-protein coding gene
	Os01g0819500	1	36671129	36671482	+	Os01t0819500-01	Similar to Ubiquitin-conjugating enzyme E2-17 kDa 11 (EC 63.2.19) (Ubiquitin- protein ligase 11) (Ubiquitin carrier protein 11)
	Os01g0819700	1	36673490	36675818	+	Os01t0819700-01	Hypothetical conserved gene
	Os01g0819800	1	36676300	36678709	+	Os01t0819800-01	Pentatricopeptide repeat domain containing protein
	Os01g0819900	1	36678850	36689035	-	Os01t0819900-01	Armadillo-like helical domain containing protein
	Os01g0820000	1	36691656	36693545	-	Os01t0820000-00	Similar to Cinnamate-4-hydroxylase
	Os01g0820400	1	36710948	36715156	+	Os01t0820400-00	Similar to WRKY22 - superfamily of TFs having WRKY and zinc finger domains
	Os01g0820750	1	36726549	36729056	-	Os01t0820750-01	Non-protein coding transcript
	Os01g0820800	1	36733512	36735285	-	Os01t0820800-01	Conserved hypothetical protein
	Os01g0821300	1	36762540	36763339	-	Os01t0821300-01	Hypothetical conserved gene
	Os01g0821600	1	36792420	36794154	+	Os01t0821600-01	WRKY transcription factor 48-like protein (WRKY transcription factor 21)

SNP	Locus (IRGSP Build 5)	Chromosome	Location (IRGSP Build 5)		DNA Strand Direction	Gene Product	Annotation
			Start (bp)	End (bp)			
	Os01g0821700	1	36794127	36797889	-	Os01t0821700-01	Similar to Chitin-binding lectin 1 precursor (PL-I)
	Os02g0150300	2	2776448	2779279	+	Os02t0150300-01	FAD-dependent pyridine nucleotide-disulphide oxidoreductase domain containing protein
	Os02g0150450	2	2780092	2782420	+	Os02t0150450-00	Similar to predicted protein
	Os02g0150500	2	2781503	2782730	-	Os02t0150500-00	Non-protein coding gene
	Os02g0150600	2	2787000	2789073	+	Os02t0150600-01	Similar to Pyridine nucleotide-disulphide oxidoreductase (Fragment)
	Os02g0150700	2	2791279	2795404	+	Os02t0150700-01	Similar to RING finger protein 12 (LIM domain interacting RING finger protein) (RING finger LIM domain-binding protein) (R-LIM) (NY-REN-43 antigen)
	Os02g0150800	2	2798814	2803579	+	Os02t0150800-01	Kelch-type beta propeller domain containing protein
S02_2877652	Os02g0150900	2	2804374	2807633	-	Os02t0150900-01	Protein of unknown function DUF1644 family protein
						Os02t0150900-02	Protein of unknown function DUF1644 family protein
	Os02g0151100	2	2816622	2824131	-	Os02t0151100-01	Hypothetical conserved gene
	Os02g0151300	2	2824486	2830255	-	Os02t0151300-01	Similar to Cellulase (Fragment)
	Os02g0151400	2	2838177	2842309	+	Os02t0151400-01	Conserved hypothetical protein
	Os02g0151600	2	2846547	2847426	+	Os02t0151600-00	Ketol-acid reductoisomerase, chloroplast precursor (EC 1.1.1.86) (Acetohydroxy-acid reductoisomerase) (Alpha-keto-beta-hydroxylacil reductoisomerase)
	Os02g0151800	2	2850257	2862112	-	Os02t0151800-01	Conserved hypothetical protein
	Os02g0152200	2	2865056	2869029	+	Os02t0152200-01	Protein of unknown function DUF1677, Oryza sativa family protein
						Os02t0152200-02	Protein of unknown function DUF1677, Oryza sativa family protein

SNP	Locus (IRGSP Build 5)	Chromosome	Location (IRGSP Build 5)		DNA Strand Direction	Gene Product	Annotation
			Start (bp)	End (bp)			
	Os02g0152300	2	2869668	2874060	+	Os02t0152300-01	Serine/threonine protein kinase-related domain containing protein
	Os02g0152400	2	2874949	2876252	+	Os02t0152400-01	Similar to Ribulose bisphosphate carboxylase (EC 4.1.1.39) (Fragment)
	Os02g0152500	2	2876557	2882175	-	Os02t0152500-01	Fibronectin, type III domain containing protein
						Os02t0152500-02	Non-protein coding transcript
	Os02g0152600	2	2898895	2903749	-	Os02t0152600-01	Protein of unknown function DUF2343 domain containing protein
	Os02g0152700	2	2904356	2906569	-	Os02t0152700-01	Conserved hypothetical protein
	Os02g0152800	2	2913663	2919107	-	Os02t0152800-01	RNA polymerase Rpb1, domain 1 containing protein
	Os02g0152900	2	2922459	2925071	-	Os02t0152900-01	Conserved hypothetical protein
	Os02g0153000	2	2928920	2932165	+	Os02t0153000-01	Protein of unknown function DUF547 domain containing protein
	Os02g0153100	2	2933950	2937377	-	Os02t0153100-00	Protein kinase, core domain containing protein
	Os02g0153200	2	2938044	2941435	-	Os02t0153200-01	Protein kinase, core domain containing protein
	Os02g0153300	2	2944659	2949243	+	Os02t0153300-01	Conserved hypothetical protein
						Os02t0153300-02	Conserved hypothetical protein
	Os02g0153400	2	2945510	2948972	-	Os02t0153400-00	Protein kinase, core domain containing protein
	Os02g0153450	2	2952127	2955764	+	Os02t0153450-01	Hypothetical gene
	Os02g0153500	2	2952422	2955600	-	Os02t0153500-00	Protein kinase, core domain containing protein
	Os02g0153600	2	2957938	2960125	+	Os02t0153600-01	Conserved hypothetical protein
	Os02g0153700	2	2958784	2962175	-	Os02t0153700-01	Serine/threonine protein kinase domain containing protein

SNP	Locus (IRGSP Build 5)	Chromosome	Location (IRGSP Build 5)		DNA Strand Direction	Gene Product	Annotation
			Start (bp)	End (bp)			
	Os02g0153900	2	2965240	2968612	-	Os02t0153900-01	Protein kinase, core domain containing protein
	Os02g0153950	2	2967242	2968573	+	Os02t0153950-00	Non-protein coding gene
	Os02g0154000	2	2972110	2975490	-	Os02t0154000-00	Protein kinase, core domain containing protein
	Os02g0154050	2	2974073	2975404	+	Os02t0154050-00	Hypothetical gene
	Os02g0571100	2	22671648	22674529	+	Os02t0571100-01	Terpenoid synthase domain containing protein
	Os02g0571300	2	22680578	22690589	-	Os02t0571300-01	Terpenoid synthase domain containing protein
	Os02g0571800	2	22713501	22724683	-	Os02t0571800-00	Terpene synthase-like domain containing protein
	Os02g0571900	2	22731595	22733235	-	Os02t0571900-01	Cytochrome P450 family protein
	Os02g0572000	2	22731821	22733340	+	Os02t0572000-01	Conserved hypothetical protein
	Os02g0572050	2	22738100	22741155	+	Os02t0572050-00	Similar to Ent-cassa-12,15-diene synthase
	Os02g0572200	2	22744365	22745676	+	Os02t0572200-01	Similar to RING-H2 finger protein ATL3I (YGHL1-C3HC4 RING fusion protein)
	Os02g0572300	2	22749914	22750960	-	Os02t0572300-00	Similar to RING-H2 finger protein ATL3B
S02_22742146	Os02g0572350	2	22754710	22755840	-	Os02t0572350-00	Hypothetical conserved gene
	Os02g0572400	2	22757664	22762562	-	Os02t0572400-01	Similar to Riboflavin biosynthesis protein ribA, chloroplast precursor [Includes: GTP cyclohydrolase II (EC 3.5.4.25); 3,4-dihydroxy-2-butanone 4- phosphate synthase (DHBP synthase)]
						Os02t0572400-02	Similar to predicted protein
	Os02g0572600	2	22776901	22778769	-	Os02t0572600-01	Protein kinase PKN/PRK1, effector domain containing protein
	Os02g0572900	2	22783712	22787118	-	Os02t0572900-01	Zinc finger, C2H2-type domain containing protein
	Os02g0573200	2	22803541	22807271	+	Os02t0573200-01	Similar to Cryptochrome 1
	Os02g0573300	2	22808050	22813504	-	Os02t0573300-01	Nucleotide-sugar transporter family protein

SNP	Locus (IRGSP Build 5)	Chromosome	Location (IRGSP Build 5)		DNA Strand Direction	Gene Product	Annotation
			Start (bp)	End (bp)			
	Os02g0573400	2	22813706	22818416	-	Os02t0573400-01	Peptidase C19, ubiquitin carboxyl-terminal hydrolase 2 family protein
	Os02g0573500	2	22822521	22829516	+	Os02t0573500-01	Similar to Monosaccharide transporter 1
	Os02g0573601	2	22827712	22829000	-	Os02t0573601-00	Hypothetical gene
	Os02g0574000	2	22837743	22839097	+	Os02t0574000-01	Similar to Monosaccharide transporter 1
	Os02g0584200	2	23356053	23358376	-	Os02t0584200-01	Conserved hypothetical protein
	Os02g0584300	2	23362356	23363235	+	Os02t0584300-01	Similar to B0403H10-OSIGBa0105A11.18 protein
	Os02g0584700	2	23377852	23378581	-	Os02t0584700-01	Heavy metal transport/detoxification protein domain containing protein
	Os02g0584800	2	23380377	23381239	-	Os02t0584800-01	Heavy metal transport/detoxification protein domain containing protein
	Os02g0584900	2	23383913	23385118	-	Os02t0584900-01	Conserved hypothetical protein
	Os02g0585100	2	23385878	23386606	-	Os02t0585100-00	Similar to ATFP4
	Os02g0585200	2	23389003	23389943	-	Os02t0585200-01	Heavy metal transport/detoxification protein domain containing protein
S02_23451362	Os02g0585566	2	23397817	23398506	-	Os02t0585566-00	Hypothetical genes
	Os02g0585600	2	23398616	23398957	+	Os02t0585600-00	Similar to H0815C01.6 protein
	Os02g0585650	2	23399224	23400338	+	Os02t0585650-01	Hypothetical gene
	Os02g0585700	2	23399567	23400253	-	Os02t0585700-00	Quinonprotein alcohol dehydrogenase-like domain containing protein
	Os02g0586000	2	23406158	23407610	-	Os02t0586000-01	Quinonprotein alcohol dehydrogenase-like domain containing protein
	Os02g0586400	2	23426443	23429110	+	Os02t0586400-01	Similar to Small GTP binding protein Rab2
	Os02g0586500	2	23429415	23432751	-	Os02t0586500-01	Similar to OSIGBa0124N08.5 protein
						Os02t0586500-02	Similar to Small nuclear ribonucleoprotein Sm D1 (snRNP core protein D1) (Sm-D1) (Sm-D autoantigen)

SNP	Locus (IRGSP Build 5)	Chromosome	Location (IRGSP Build 5)		DNA Strand Direction	Gene Product	Annotation
			Start (bp)	End (bp)			
	Os02g0586800	2	23444500	23447005	+	Os02t0586800-01	Tetratricopeptide-like helical domain containing protein
	Os02g0586900	2	23449381	23450158	+	Os02t0586900-01	Hypothetical conserved gene
						Os02t0586900-02	Similar to Glycine rich protein (Fragment)
	Os02g0587000	2	23453271	23454177	+	Os02t0587000-01	Similar to Glycine rich protein (Fragment)
	Os02g0587800	2	23477956	23478929	+	Os02t0587800-01	Virulence factor, pectin lyase fold family protein
	Os02g0588250	2	23493017	23497459	+	Os02t0588250-01	Hypothetical gene
	Os02g0588300	2	23493455	23498440	-	Os02t0588300-01	Similar to Fimbrin 1 (AtFIM1)
	Os02g0588500	2	23514275	23519265	+	Os02t0588500-01	Hypothetical conserved gene
						Os02t0588500-02	PLC-like phosphodiesterase, TIM beta/alpha-barrel domain domain containing protein
	Os02g0588550	2	23517220	23520001	-	Os02t0588550-00	Hypothetical gene
	Os02g0588600	2	23519607	23521026	+	Os02t0588600-01	Conserved hypothetical protein
	Os02g0588700	2	23523560	23524567	-	Os02t0588700-01	Conserved hypothetical protein
	Os03g0183800	3	4396243	4401936	+	Os03t0183800-01	Similar to Leucine-rich repeat transmembrane protein kinase 1 (Fragment)
						Os03t0183800-02	Similar to Leucine-rich repeat transmembrane protein kinase 1 (Fragment)
	Os03g0183900	3	4402061	4404967	-	Os03t0183900-01	Similar to Plasma membrane H+-ATPase
	Os03g0184000	3	4409088	4413503	+	Os03t0184000-00	Similar to Phytoene desaturase (Fragment)
S03_4501040	Os03g0184050	3	4413613	4413777	+	Os03t0184050-01	Non-protein coding transcript
	Os03g0184100	3	4415652	4418089	+	Os03t0184100-01	Hypothetical protein
	Os03g0184201	3	4419238	4419993	+	Os03t0184201-00	Hypothetical conserved gene
	Os03g0184300	3	4427836	4433159	+	Os03t0184300-02	Glycosyl transferase, family 8 protein
						Os03t0184300-01	Glycosyl transferase, family 8 protein
	Os03g0184400	3	4433558	4436811	+	Os03t0184400-01	Leucine-rich repeat, plant specific containing protein

SNP	Locus (IRGSP Build 5)	Chromosome	Location (IRGSP Build 5)		DNA Strand Direction	Gene Product	Annotation
			Start (bp)	End (bp)			
	Os03g0184500	3	4436780	4438872	-	Os03t0184500-01	Transcriptional factor B3 family protein
	Os03g0184550	3	4440958	4442826	+	Os03t0184550-01	Similar to Dihydroflavonol-4-reductase
	Os03g0184600	3	4443013	4445464	+	Os03t0184600-01	NAD(P)-binding domain containing protein
	Os03g0184700	3	4445726	4448161	-	Os03t0184700-01	Origin recognition complex subunit 2 (Origin recognition complex2)
	Os03g0185000	3	4463129	4464268	+	Os03t0185000-00	Similar to Chloroplast serine acetyltransferase
	Os03g0185200	3	4469565	4475126	-	Os03t0185200-01	Pentatricopeptide repeat domain containing protein
	Os03g0185300	3	4470000	4470948	-	Os03t0185300-01	Hypothetical protein
	Os03g0185400	3	4478674	4480245	-	Os03t0185400-01	Protein of unknown function DUF1677, Oryza sativa family protein
	Os03g0185450	3	4482357	4483654	-	Os03t0185450-00	Non-protein coding gene
	Os03g0185500	3	4485360	4488498	+	Os03t0185500-01	Conserved hypothetical protein
	Os03g0185600	3	4488693	4489508	+	Os03t0185600-01	Conserved hypothetical protein
	Os03g0185700	3	4491082	4493046	-	Os03t0185700-01	Transferase family protein
	Os03g0185800	3	4493722	4494489	+	Os03t0185800-01	Conserved hypothetical protein
	Os03g0186100	3	4495732	4499822	+	Os03t0186100-01	Similar to Uroporphyrinogen III synthase
	Os03g0186500	3	4512396	4517519	+	Os03t0186500-01	Myoactive tetradecapeptides family protein
						Os03t0186500-02	Hypothetical conserved gene
						Os03t0186500-03	Hypothetical conserved gene
	Os03g0186600	3	4518403	4524690	-	Os03t0186600-01	Transcription factor, MADS-box domain containing protein
	Os03g0186800	3	4531884	4535582	+	Os03t0186800-01	Modifier of rudimentary, Modr family protein
	Os03g0186900	3	4540349	4542090	+	Os03t0186900-01	RICE ANTHER DOWN-REGULATED BY CHILLING 1, aspartic protease 25 (Radc1)
						Os03t0186900-02	RICE ANTHER DOWN-REGULATED BY CHILLING 1, aspartic protease 25 (Radc1)

SNP	Locus (IRGSP Build 5)	Chromosome	Location (IRGSP Build 5)		DNA Strand Direction	Gene Product	Annotation
			Start (bp)	End (bp)			
	Os03g0186950	3	4542172	4544070	-	Os03t0186950-01	Similar to CUTA
	Os03g0187000	3	4544304	4546964	+	Os03t0187000-01	Conserved hypothetical protein
	Os03g0187100	3	4554658	4558468	+	Os03t0187100-00	Hypothetical conserved gene
	Os03g0187200	3	4558622	4558827	+	Os03t0187200-01	Non-protein coding transcript
	Os03g0187300	3	4560078	4568080	+	Os03t0187300-01	Tetratricopeptide-like helical domain containing protein
	Os03g0187350	3	4568624	4570036	-	Os03t0187350-01	Hypothetical protein
	Os03g0187400	3	4572146	4574548	+	Os03t0187400-01	Zinc finger, FYVE/PHD-type domain containing protein
	Os03g0187500	3	4583425	4584585	-	Os03t0187500-01	Leucine-rich repeat, cysteine-containing subtype containing protein
						Os03t0187500-02	Similar to Leucine Rich Repeat family protein, expressed
	Os03g0187550	3	4591217	4593342	-	Os03t0187550-01	Hypothetical gene
	Os03g0187600	3	4591487	4593795	+	Os03t0187600-01	GRAM domain containing protein
	Os03g0187700	3	4595106	4597255	+	Os03t0187700-01	Target SNARE coiled-coil region domain containing protein
	Os03g0187800	3	4597922	4599428	-	Os03t0187800-01	Protein of unknown function DUF250 domain containing protein
	Os03g0815400	3	35073328	35075216	+	Os03t0815400-01	Ribosomal protein L17-like protein
	Os03g0815700	3	35084986	35089286	+	Os03t0815700-01	K Homology domain containing protein
S03_35171822	Os03g0815800	3	35090980	35092199	-	Os03t0815800-01	Similar to Ethylene-responsive transcription factor 5 (Ethylene-responsive element binding factor 5) (EREBP-5) (AtERF5)
						Os03t0815800-02	Similar to Ethylene-responsive transcription factor 5 (Ethylene-responsive element binding factor 5) (EREBP-5) (AtERF5)
						Os03t0815800-03	Hypothetical conserved gene
	Os03g0815900	3	35093775	35096539	-	Os03t0815900-01	Similar to DNA-directed RNA polymerase
	Os03g0816000	3	35097428	35100438	-	Os03t0816000-01	Hypothetical conserved gene

SNP	Locus (IRGSP Build 5)	Chromosome	Location (IRGSP Build 5)		DNA Strand Direction	Gene Product	Annotation
			Start (bp)	End (bp)			
	Os03g0816100	3	35101013	35104820	-	Os03t0816100-01	Similar to Protein kinase
	Os03g0816200	3	35113959	35116649	+	Os03t0816200-01	Ribosomal protein S26e domain containing protein
	Os03g0816300	3	35118496	35121232	-	Os03t0816300-01	Conserved hypothetical protein
	Os03g0816400	3	35126077	35130682	-	Os03t0816400-01	GTP-binding protein, HSR1-related domain containing protein
						Os03t0816400-02	GTP-binding protein, HSR1-related domain containing protein
	Os03g0816500	3	35132995	35136955	+	Os03t0816500-01	Hypothetical conserved gene
	Os03g0816600	3	35137023	35139932	+	Os03t0816600-01	Pentatricopeptide repeat domain containing protein
	Os03g0816700	3	35140713	35141968	-	Os03t0816700-01	Protein of unknown function DUF567 family protein
	Os03g0816800	3	35143645	35144761	-	Os03t0816800-01	Protein of unknown function DUF567 family protein
	Os03g0816900	3	35156407	35162120	+	Os03t0816900-01	Conserved hypothetical protein
	Os03g0816950	3	35158828	35164912	-	Os03t0816950-01	Conserved hypothetical protein
	Os03g0817000	3	35163522	35165411	+	Os03t0817000-01	Actin-binding WH2 domain containing protein
	Os03g0817100	3	35166032	35167260	-	Os03t0817100-00	Uncharacterized protein family UPF0497, trans-membrane plant domain containing protein
	Os03g0817200	3	35169910	35171616	+	Os03t0817200-01	Amino acid transporter, transmembrane domain containing protein
	Os03g0817400	3	35186320	35191217	+	Os03t0817400-00	Hypothetical gene
	Os03g0817500	3	35188408	35191331	-	Os03t0817500-01	Protein of unknown function DUF231, plant domain containing protein
	Os03g0817700	3	35203685	35207293	+	Os03t0817700-00	Hypothetical conserved gene
	Os03g0817800	3	35209831	35213346	-	Os03t0817800-01	Protein of unknown function DUF231 domain containing protein

SNP	Locus (IRGSP Build 5)	Chromosome	Location (IRGSP Build 5)		DNA Strand Direction	Gene Product	Annotation
			Start (bp)	End (bp)			
						Os03t0817800-02	Conserved hypothetical protein
	Os03g0817900	3	35219067	35223930	-	Os03t0817900-01	Protein of unknown function DUF231, plant domain containing protein
	Os03g0818000	3	35225699	35231183	+	Os03t0818000-00	Similar to myosin-like protein
	Os03g0818050	3	35231792	35232030	+	Os03t0818050-00	Hypothetical conserved gene
	Os03g0818100	3	35233305	35238682	+	Os03t0818100-01	Histidine acid phosphatase family protein
						Os03t0818100-02	Similar to Multiple inositol polyphosphate phosphatase PhyIIa2
	Os03g0818200	3	35238836	35241760	+	Os03t0818200-01	NAD(P)-binding domain containing protein
	Os03g0818300	3	35241696	35245284	-	Os03t0818300-01	Zinc finger, RING/FYVE/PHD-type domain containing protein
	Os03g0818400	3	35245727	35247964	-	Os03t0818400-01	Similar to 40S ribosomal protein S23 (S12)
	Os03g0818700	3	35249148	35251278	+	Os03t0818700-00	Hypothetical conserved gene
	Os03g0818800	3	35256742	35260883	+	Os03t0818800-00	Similar to APETALA2-like protein
S04_5091349	Os04g0173800	4	4993702	4994796	-	Os04t0173800-01	Lectin precursor (Agglutinin)
	Os04g0174200	4	5046921	5049847	-	Os04t0174200-01	Conserved hypothetical protein
	Os04g0174800	4	5100549	5101413	-	Os04t0174800-01	Similar to predicted protein
	Os04g0174900	4	5109620	5112965	+	Os04t0174900-01	Similar to Acyl-protein thioesterase 2
	Os04g0175000	4	5111924	5115319	-	Os04t0175000-01	Conserved hypothetical protein
						Os04t0175000-02	Non-protein coding transcript
	Os04g0175300	4	5123820	5130704	-	Os04t0175300-01	Conserved hypothetical protein
						Os04t0175300-02	Conserved hypothetical protein
	Os04g0175400	4	5124185	5124919	+	Os04t0175400-01	Conserved hypothetical protein
	Os04g0175500	4	5140682	5141977	-	Os04t0175500-00	Transferase family protein
	Os04g0175600	4	5150540	5156065	+	Os04t0175600-01	Similar to 0-methyltransferase (EC 2.1.1.6) (Fragment)
	Os04g0175750	4	5156298	5159054	-	Os04t0175750-01	Non-protein coding transcript

SNP	Locus (IRGSP Build 5)	Chromosome	Location (IRGSP Build 5)		DNA Strand Direction	Gene Product	Annotation
			Start (bp)	End (bp)			
S04_13964201	Os04g0175900	4	5167084	5175078	+	Os04t0175900-01	Winged helix repressor DNA-binding domain containing protein
	Os04g0176200	4	5178543	5183597	-	Os04t0176200-00	Winged helix repressor DNA-binding domain containing protein
	Os04g0303950	4	13862920	13868523	-	Os04t0303950-01	Exonuclease, RNase T and DNA polymerase III domain containing protein
	Os04g0304000	4	13870803	13877123	-	Os04t0304000-01	Conserved hypothetical protein
	Os04g0304200	4	13893733	13906921	-	Os04t0304200-01	Similar to Nonphototropic hypocotyl protein 1 (EC 2.7.1.37) (Phototropin)
	Os04g0304400	4	13924782	13927956	+	Os04t0304400-01	Similar to MADS-box protein AGL16-II
	Os04g0305700	4	14023544	14025346	+	Os04t0305700-01	UDP-glucuronosyl/UDP-glucosyltransferase family protein
						Os04t0305700-02	UDP-glucuronosyl/UDP-glucosyltransferase family protein
	Os04g0306300	4	14048640	14049521	-	Os04t0306300-00	Thaumatin, pathogenesis-related family protein
	Os04g0306400	4	14054352	14055530	+	Os04t0306400-01	Ribose 5-phosphate isomerase family protein
S06_6057367	Os06g0217300	6	5951600	5962310	-	Os06t0217300-01	Similar to Transcription factor MADS55
	Os06g0217500	6	5976402	5979738	-	Os06t0217500-01	MED6 mediator family protein
	Os06g0217600	6	5981682	5983397	-	Os06t0217600-01	Hypothetical protein
	Os06g0217700	6	6006467	6009098	-	Os06t0217700-01	Conserved hypothetical protein
	Os06g0217900	6	6018598	6020842	+	Os06t0217900-01	Cyclin D domain containing protein
	Os06g0218000	6	6026824	6028450	+	Os06t0218000-00	Similar to pre-mRNA-splicing factor prp45
	Os06g0218150	6	6035740	6045263	-	Os06t0218150-00	Hypothetical conserved gene
	Os06g0218200	6	6045736	6049763	-	Os06t0218200-01	Zinc finger, SWIM-type domain containing protein
	Os06g0218300	6	6057842	6059542	-	Os06t0218300-01	Conserved hypothetical protein
	Os06g0218500	6	6084460	6096661	-	Os06t0218500-01	MCM family protein
						Os06t0218500-02	MCM family protein

SNP	Locus (IRGSP Build 5)	Chromosome	Location (IRGSP Build 5)		DNA Strand Direction	Gene Product	Annotation
			Start (bp)	End (bp)			
						Os06t0218500-03	Non-protein coding transcript
	Os06g0218600	6	6085860	6087131	+	Os06t0218600-01	Cupredoxin domain containing protein
	Os06g0218800	6	6100062	6101056	+	Os06t0218800-01	Conserved hypothetical protein
						Os06t0218800-02	Conserved hypothetical protein
	Os06g0218900	6	6101186	6103365	-	Os06t0218900-00	Similar to predicted protein
	Os06g0219400	6	6145640	6149664	+	Os06t0219400-00	Similar to predicted protein
	Os06g0219500	6	6151297	6152503	-	Os06t0219500-00	Similar to Heat shock 22 kDa protein, mitochondrial precursor
	Os06g0219600	6	6154941	6158842	+	Os06t0219600-01	Similar to Poly(A)-binding protein II-like
S07_22868199	Os07g0555000	7	22767847	22770288	+	Os07t0555000-01	F-box domain, Skp2-like domain containing protein
						Os07t0555000-02	F-box domain protein, Regulation of seed development (OsFbox394)
	Os07g0555100	7	22770984	22773832	+	Os07t0555100-01	Conserved hypothetical protein
	Os07g0555200	7	22775119	22783287	+	Os07t0555200-01	Hypothetical conserved gene
						Os07t0555200-02	Similar to Eukaryotic translation initiation factor 4G
						Os07t0555200-03	Similar to predicted protein
						Os07t0555200-04	Similar to predicted protein
						Os07t0555200-05	Similar to predicted protein
	Os07g0555300	7	22785376	22788081	+	Os07t0555300-01	Conserved hypothetical protein
						Os07t0555300-02	Conserved hypothetical protein
	Os07g0555400	7	22788660	22790556	+	Os07t0555400-01	Conserved hypothetical protein
	Os07g0556000	7	22829680	22831881	-	Os07t0556000-01	Cyclin D domain containing protein
	Os07g0556200	7	22844565	22847048	+	Os07t0556200-01	Rieske [2Fe-2S] region domain containing protein
	Os07g0556300	7	22847594	22849265	-	Os07t0556300-01	Octicosapeptide/Phox/Bem1p domain containing protein

SNP	Locus (IRGSP Build 5)	Chromosome	Location (IRGSP Build 5)		DNA Strand Direction	Gene Product	Annotation
			Start (bp)	End (bp)			
	Os07g0556500	7	22857247	22863794	-	Os07t0556500-01	Hypothetical conserved gene
	Os07g0556800	7	22882458	22883494	-	Os07t0556800-01	Ribosome-inactivating protein family protein
	Os07g0557100	7	22891185	22894709	-	Os07t0557100-01	Delayed-early response protein/equilibrative nucleoside transporter family protein
						Os07t0557100-02	Delayed-early response protein/equilibrative nucleoside transporter family protein
	Os07g0557200	7	22896979	22900477	-	Os07t0557200-01	Delayed-early response protein/equilibrative nucleoside transporter family protein
	Os07g0557400	7	22904083	22906648	-	Os07t0557400-01	Delayed-early response protein/equilibrative nucleoside transporter family protein
	Os07g0557500	7	22907815	22912990	+	Os07t0557500-01	WRC domain containing protein
						Os07t0557500-02	Hypothetical conserved gene
	Os07g0557601	7	22916435	22918647	+	Os07t0557601-00	Hypothetical gene
	Os07g0557700	7	22916435	22918700	-	Os07t0557700-00	Conserved hypothetical protein
	Os07g0558000	7	22927907	22933774	-	Os07t0558000-01	ABC-1 domain containing protein
						Os07t0558000-02	ABC-1 domain containing protein
	Os07g0558100	7	22953894	22955467	-	Os07t0558100-00	Similar to Myb-related transcription factor LBM1
	Os07g0558200	7	22964018	22968834	-	Os07t0558200-01	Inositol monophosphatase family protein
						Os07t0558200-02	Similar to predicted protein
S08_8532874	Os08g0239300	8	8436327	8444920	+	Os08t0239300-01	Dienelactone hydrolase domain containing protein
	Os08g0239900	8	8463917	8468525	+	Os08t0239900-00	Hypothetical conserved gene
	Os08g0240000	8	8471525	8474593	-	Os08t0240000-01	Similar to STF-1 (Fragment)
						Os08t0240000-02	Similar to STF-1 (Fragment)
	Os08g0240200	8	8478587	8480493	+	Os08t0240200-01	Conserved hypothetical protein
	Os08g0240300	8	8483509	8483781	-	Os08t0240300-00	Hypothetical conserved gene
	Os08g0240500	8	8487371	8488592	+	Os08t0240500-00	Similar to OSIGBa0118P15.7 protein

SNP	Locus (IRGSP Build 5)	Chromosome	Location (IRGSP Build 5)		DNA Strand Direction	Gene Product	Annotation
			Start (bp)	End (bp)			
	Os08g0240600	8	8496295	8499130	-	Os08t0240600-00	Similar to predicted protein
	Os08g0240800	8	8507467	8517594	+	Os08t0240800-01	Similar to Actin filament bundling protein P-115-ABP
	Os08g0240966	8	8526772	8527657	+	Os08t0240966-00	Non-protein coding transcript
	Os08g0241300	8	8569834	8574242	+	Os08t0241300-01	Conserved hypothetical protein
	Os08g0241400	8	8580996	8583062	+	Os08t0241400-00	Similar to protein binding protein
	Os08g0241600	8	8588249	8593476	-	Os08t0241600-01	Similar to apospory-associated protein C
	Os08g0241800	8	8612750	8614703	-	Os08t0241800-01	Similar to Plasma membrane H+-ATPase (EC 3.6.1.3)
	Os08g0241900	8	8614724	8616850	+	Os08t0241900-01	Conserved hypothetical protein
	Os08g0477600	8	23639870	23642178	-	Os08t0477600-01	Conserved hypothetical protein
	Os08g0477700	8	23645561	23646751	+	Os08t0477700-00	Molecular chaperone, heat shock protein, Hsp40, DnaJ domain containing protein
	Os08g0477800	8	23649847	23654389	+	Os08t0477800-01	PWWP domain containing protein
	Os08g0477900	8	23660588	23661393	+	Os08t0477900-00	Helix-loop-helix DNA-binding domain containing protein
	Os08g0478000	8	23663572	23664312	+	Os08t0478000-01	Similar to mucin-2
						Os08t0478000-02	Cell surface antigen domain containing protein
S08_23740235	Os08g0478100	8	23664753	23667093	-	Os08t0478100-01	Uncharacterised protein family UPF0029, N-terminal domain containing protein
	Os08g0478200	8	23667301	23670150	-	Os08t0478200-01	Similar to ATP synthase D chain, mitochondrial (EC 3.6.3.14)
	Os08g0478466	8	23677359	23678692	-	Os08t0478466-01	Protein of unknown function DUF296 domain containing protein
	Os08g0478500	8	23681707	23689137	-	Os08t0478500-01	Peptidase C19, ubiquitin carboxyl-terminal hydrolase 2 family protein
	Os08g0478700	8	23701115	23702402	-	Os08t0478700-01	Similar to Mitochondrial uncoupling protein (Fragment)

SNP	Locus (IRGSP Build 5)	Chromosome	Location (IRGSP Build 5)		DNA Strand Direction	Gene Product	Annotation
			Start (bp)	End (bp)			
	Os08g0478800	8	23711297	23716562	-	Os08t0478800-01	Phosphoglucose isomerase (PGI) family protein
	Os08g0479300	8	23736014	23739078	+	Os08t0479300-01	Cyclin, A/B/D/E domain containing protein
	Os08g0479400	8	23751497	23753387	-	Os08t0479400-01	Similar to Hydroxyproline-rich glycoprotein DZ-HRGP precursor
	Os08g0480000	8	23800637	23805525	+	Os08t0480000-01	Multi antimicrobial extrusion protein MatE family protein
	Os08g0480050	8	23803232	23804441	-	Os08t0480050-00	Hypothetical gene
	Os08g0480100	8	23805768	23810254	-	Os08t0480100-01	Signal recognition particle receptor, alpha subunit, N-terminal domain containing protein
						Os08t0480100-02	Similar to predicted protein
	Os08g0480200	8	23811620	23815252	-	Os08t0480200-01	2OG-Fe(II) oxygenase domain containing protein
	Os08g0480400	8	23818719	23820298	-	Os08t0480400-01	Similar to cupin, RmlC-type
	Os08g0480500	8	23829492	23834982	-	Os08t0480500-01	Surfeit locus 5 family protein
	Os08g0480800	8	23838342	23840513	+	Os08t0480800-01	Similar to TaWIN2
	Os08g0480901	8	23838463	23840093	-	Os08t0480901-00	Non-protein coding gene
S08_27534174	Os08g0545900	8	27433299	27434909	-	Os08t0545900-00	Multi antimicrobial extrusion protein MatE family protein
	Os08g0546100	8	27445030	27446488	-	Os08t0546100-00	Similar to protein binding protein
	Os08g0546300	8	27452170	27452821	+	Os08t0546300-01	Conserved hypothetical protein
	Os08g0546400	8	27453143	27458828	-	Os08t0546400-01	UBX domain containing protein
	Os08g0546467	8	27453248	27455414	+	Os08t0546467-00	Hypothetical gene
	Os08g0546533	8	27462683	27462888	+	Os08t0546533-01	Non-protein coding transcript
	Os08g0546600	8	27462939	27463641	+	Os08t0546600-01	Non-protein coding transcript
	Os08g0546700	8	27464057	27466944	-	Os08t0546700-01	Peptidase S54, rhomboid domain containing protein

SNP	Locus (IRGSP Build 5)	Chromosome	Location (IRGSP Build 5)		DNA Strand Direction	Gene Product	Annotation
			Start (bp)	End (bp)			
	Os08g0546800	8	27470877	27472475	-	Os08t0546800-01	Similar to Heat stress transcription factor B-2b
	Os08g0546900	8	27481309	27484089	-	Os08t0546900-01	Similar to NC domain-containing protein
	Os08g0547000	8	27485953	27489082	+	Os08t0547000-01	RNA recognition motif, RNP-1 domain containing protein
						Os08t0547100-01	Similar to 6-phosphogluconolactonase
	Os08g0547100	8	27491449	27494443	+	Os08t0547100-02	6-phosphogluconolactonase domain containing protein
						Os08t0547100-03	6-phosphogluconolactonase domain containing protein
	Os08g0547200	8	27496984	27501885	+	Os08t0547200-01	RabGAP/TBC domain containing protein
	Os08g0547300	8	27508638	27510840	-	Os08t0547300-01	Similar to Cytochrome P450 78A1 (EC 1.14.-.-) (CYPLXXVIII)
	Os08g0547500	8	27523131	27530443	-	Os08t0547500-01	Similar to Kinesin-like protein NACK1
						Os08t0547500-02	Hypothetical conserved gene
	Os08g0547600	8	27546432	27547769	-	Os08t0547600-00	Protein of unknown function DUF702 family protein
	Os08g0547800	8	27561752	27563047	-	Os08t0547800-01	Alpha/beta hydrolase fold-3 domain containing protein
	Os08g0547900	8	27567067	27569064	-	Os08t0547900-01	Cytochrome P450 family protein
	Os08g0548000	8	27574403	27576803	-	Os08t0548000-01	Myb transcription factor domain containing protein
	Os08g0548200	8	27585236	27589009	+	Os08t0548200-01	Similar to ER lumen protein retaining receptor-like protein
						Os08t0548200-02	Similar to ER lumen protein retaining receptor-like protein
	Os08g0548300	8	27590333	27592977	+	Os08t0548300-01	Zinc finger, RING/FYVE/PHD-type domain containing protein
	Os08g0548400	8	27595622	27596482	+	Os08t0548400-00	Similar to chaperone protein dnaJ 11
	Os08g0548500	8	27599201	27601559	+	Os08t0548500-01	Armadillo-like helical domain containing protein

SNP	Locus (IRGSP Build 5)	Chromosome	Location (IRGSP Build 5)		DNA Strand Direction	Gene Product	Annotation
			Start (bp)	End (bp)			
	Os08g0548600	8	27602813	27604120	-	Os08t0548600-01	Hypothetical conserved gene
	Os08g0548650	8	27607884	27608606	-	Os08t0548650-00	Hypothetical gene
	Os08g0548700	8	27608999	27609455	+	Os08t0548700-01	Hypothetical conserved gene
	Os08g0548900	8	27621334	27624731	+	Os08t0548900-01	Similar to Proteasome subunit alpha type 7 (EC 3.4.25.1) (20S proteasome alpha subunit D) (20S proteasome subunit alpha-4)
S11_23264123	Os11g0562600	11	23163989	23165909	-	Os11t0562600-00	Hypothetical conserved gene
	Os11g0564800	11	23230626	23237317	-	Os11t0564800-01	Conserved hypothetical protein
	Os11g0565000	11	23240458	23243529	+	Os11t0565000-01	Leucine-rich repeat, N-terminal domain containing protein
	Os11g0565300	11	23245791	23248104	-	Os11t0565300-00	Similar to Protein kinase domain containing protein
	Os11g0565400	11	23250582	23255773	-	Os11t0565400-01	Similar to RING finger family protein
	Os11g0566800	11	23302382	23304170	+	Os11t0566800-01	Similar to Bibenzyl synthase (EC 2.3.1.-)
	Os11g0567500	11	23338723	23340255	-	Os11t0567500-00	Hypothetical conserved gene
	Os11g0567600	11	23345348	23348453	+	Os11t0567600-01	Conserved hypothetical protein
	Os11g0567800	11	23356364	23358650	+	Os11t0567800-01	Similar to HcrVf2 protein
S12_7883496	Os12g0240850	12	7805688	7805999	+	Os12t0240850-00	Similar to POT family protein
	Os12g0240875	12	7806031	7806357	+	Os12t0240875-00	TGF-beta receptor, type I/II extracellular region domain containing protein
	Os12g0241100	12	7827813	7832546	-	Os12t0241100-01	Iojap-related protein family protein
	Os12g0242100	12	7881791	7882754	-	Os12t0242100-01	Similar to Glycine-rich cell wall structural protein 1 precursor
	Os12g0242500	12	7899882	7901405	+	Os12t0242500-01	Conserved hypothetical protein
	Os12g0242700	12	7923115	7926149	+	Os12t0242700-01	Similar to 3-oxoacyl-[acyl-carrier-protein] reductase 1, chloroplast precursor (EC 1.1.1.100) (3-ketoacyl-acyl carrier protein reductase 1) (Beta- keto acyl-carrier protein reductase 1)

SNP	Locus (IRGSP Build 5)	Chromosome	Location (IRGSP Build 5)		DNA Strand Direction	Gene Product	Annotation
			Start (bp)	End (bp)			
	Os12g0242800	12	7927082	7928048	+	Os12t0242800-01	TRAM, LAG1 and CLN8 homology domain containing protein
	Os12g0242900	12	7929142	7932434	-	Os12t0242900-01	DNA polymerase alpha, subunit B domain containing protein
	Os12g0243050	12	7939209	7942555	-	Os12t0243050-01	Hypothetical gene
	Os12g0243100	12	7943410	7943793	+	Os12t0243100-00	Hypothetical conserved gene
